# The Assessment of Medication Effects in Omicron Patients through MADM Approach Based on Distance Measures of Interval-Valued Fuzzy Hypersoft Set

**DOI:** 10.3390/bioengineering9110706

**Published:** 2022-11-17

**Authors:** Muhammad Arshad, Muhammad Saeed, Atiqe Ur Rahman, Dilovan Asaad Zebari, Mazin Abed Mohammed, Alaa S. Al-Waisy, Marwan Albahar, Mohammed Thanoon

**Affiliations:** 1 Department of Mathematics, University of Management and Technology, Lahore 54000, Pakistan; 2 Department of Computer Science, College of Science, Nawroz University, Duhok 42001, Iraq; 3 College of Computer Science and Information Technology, University of Anbar, Anbar 31001, Iraq; 4 Computer Technologies Engineering Department, Information Technology College, Imam Ja’afar Al-Sadiq University, Baghdad 10001, Iraq; 5 Department of Computer Science, Umm Al Qura University, Mecca 24211, Saudi Arabia

**Keywords:** uncertainties, monotone pattern, medication, interval-valued fuzzy set, hypersoft set, interval-valued fuzzy hypersoft set

## Abstract

Omicron, so-called COVID-2, is an emerging variant of COVID-19 which is proved to be the most fatal amongst the other variants such as alpha, beta and gamma variants (α, β, γ variants) due to its stern and perilous nature. It has caused hazardous effects globally in a very short span of time. The diagnosis and medication of Omicron patients are both challenging undertakings for researchers (medical experts) due to the involvement of various uncertainties and the vagueness of its altering behavior. In this study, an algebraic approach, interval-valued fuzzy hypersoft set (iv-FHSS), is employed to assess the conditions of patients after the application of suitable medication. Firstly, the distance measures between two iv-FHSSs are formulated with a brief description some of its properties, then a multi-attribute decision-making framework is designed through the proposal of an algorithm. This framework consists of three phases of medication. In the first phase, the Omicron-diagnosed patients are shortlisted and an iv-FHSS is constructed for such patients and then they are medicated. Another iv-FHSS is constructed after their first medication. Similarly, the relevant iv-FHSSs are constructed after second and third medications in other phases. The distance measures of these post-medication-based iv-FHSSs are computed with pre-medication-based iv-FHSS and the monotone pattern of distance measures are analyzed. It is observed that a decreasing pattern of computed distance measures assures that the medication is working well and the patients are recovering. In case of an increasing pattern, the medication is changed and the same procedure is repeated for the assessment of its effects. This approach is reliable due to the consideration of parameters (symptoms) and sub parameters (sub symptoms) jointly as multi-argument approximations.

## 1. Introduction

According to World Health Organization (WHO) [1], there are approximately 0.5 billion confirmed cases of COVID-19 and more than 6 million deaths reported as of 12 am coordinated universal time (UTC) 15 April 2022. This disease has been spreading rapidly for more than two and a half years. COVID-2 was recognized at the end of 2019 and a variety of other variations arose. On the basis of their origin and exposure, WHO has classified these variants into three main groups for screening and exploration purposes, which are named as variants under monitoring (VM), variants of interest (VI) and variants of concern (VC). Variants α,β,γ,δ are placed in the category of VC [2]. These variants are a major cause of deaths across the globe. In the last week of November 2021, Omicron arose as the fifth variant of VC, as declared by WHO, and has rapidly increased.

Fuzzy set (f-set) [3], soft set (s-set) [4] and their hybridized model fuzzy soft set (fs-set) [5] are considered as suitable models to deal with uncertainties and vagueness in data. The f-set employs a membership function for assigning fuzzy value within [0,1] to each element in the sample universe, whereas fs-set connects an approximate function to equip an f-set with a parametrization tool so that each member in the set of parameters may be parameterized with a fuzzy membership grade. Yang et al. [6] conceptualized an interval-valued fuzzy soft set (ivfs-set) to tackle the interval nature of data by intermingling a interval-valued fuzzy set (ivf-set) [7] and s-set. Researchers Peng and Garg [8], Chetia and Das [9], Ma et al. [10,11] and Qin and Ma [12] made marvelous contributions concerning real-world applications of ivfs-sets. By adjoining the concepts of ivfs-set and multi-fuzzy soft set (mFS-set) [13,14,15], Zhou et al. [16] characterized an interval-valued multi-fuzzy soft set (IVmFS-set). A single set of parameters are utilized in s-set-like models while dealing with the uncertain and vague nature of data but there are some situations when the considered parameters are not adequate for tackling decision-making problems. These parameters need further categorization into their respective subclasses. The S-set literature is not sufficient to manage such situations; therefore, Smarandache [17] introduced a new concept, hypersoft set (hs-set), as an extension of s-set. It manages the inadequacy of s-set by utilizing a new mapping known as multi-argument approximate mapping (maa-mapping). In this mapping, parametric tuples are taken as the domain and the power set of the universe as its codomain. In this way, it is very appropriate to say that hs-set is more flexible and reliable as compared to s-set due to the addition of maa-mapping. Saeed et al. [18] introduced various elementary properties and operations of hs-set to ensure the utilization of hs-set in solving real-world problems and explained them with the provision of detailed examples. Rahman et al. [19] transformed the classical concept of convexity and concavity under the hs-set environment. They [20] also managed the roughness of data through the development of a rough hs-set and discussed some of its operational properties. The expert systems for hs-set environments to deal with the situations which demand the multi-decisive opinions of experts in decision-making systems were developed by Kamacı [21] and Ihsan et al. [22,23]. Martin and Smarandache [24] broadened the concept to a plithogenic hs-set and then introduced its graphical versions. Rahman et al. [25] investigated the parametrization of fhs-set and explored its various operational results. Yolcu and Oztürk [26] developed fuzzy hs-set (fhs-set) as a hybridization of hs-set and fuzzy set and discussed its various basic operational properties. Saeed et al. [27] and Ahsan et al. [28] conferred multi-criteria decision-making problems of a complex multi fhs-set and complex fhs-set. Rahman et al. [29] discussed several variants in fhs-set under the umbrella of convexity and concavity and explored its various set-theoretic properties and operations. Debnath [30] solved decision-making problems by developing and applying different weightage operators of the fhs-set. Bhandari and Pal [31] introduced new informative measure for discrimination between two f-sets. Lee et al. [32] developed a design of similarity and dissimilarity measures for fuzzy sets on the basis of distance measure. Xindong and Yong [33] developed distance measure, entropy measure, similarity measure, inclusion measure and the subset-hood measure of ivfs-set. Khalid and Abbas [34] discussed distance measures for interval-valued intuitionistic fuzzy soft sets (ivifs-sets).

The existing literature on s-set, fs-set, hs-set and fhs-set is unable to tackle the following situations collectively:

1. A situation which demands the further categorization of attributes into their relative attributive values in the form of non-overlapping sets;
2. A situation which has a lot of data with its systematic design as interval-valued settings;
3. A situation which involves more than one distance measure for decision making;
4. A situation which has a pictorial representation of the improvement of the patient.

The significant contributions of the paper are outlined as:

1. An algebraic structure, interval-valued fuzzy hypersoft set (iv-FHSS), is employed to assess the conditions of Omicron patients after applying appropriate medication.
2. A multi-attribute decision-making framework is designed through the proposal of an algorithm based on the distance measures between two iv-FHSSs.
3. The proposed framework consists of three phases of medication. The Omicron-diagnosed patients are shortlisted and an iv-FHSS is constructed for such patients and then they are medicated in the first phase. Another iv-FHSS is constructed after their first medication. Similarly, the relevant iv-FHSSs are constructed after second and third medications in other phases. The distance measures of these post-medication-based iv-FHSSs are computed with pre-medication-based iv-FHSS and the monotone pattern of distance measures are analyzed.
4. It is observed that a decreasing pattern of computed distance measures assures that the medication is working well and the patients are recovering. In case of an increasing pattern, the medication is changed and the same procedure is repeated for the assessment of its effects. This approach is reliable due to the consideration of parameters (symptoms) and sub-parameters (sub-symptoms) jointly as multi-argument approximations.

The rest of the paper is organized as follows: Section 2 includes the basic notions of f-set, s-set, hs-set and ivf-set, etc. Section 3 presents some new operations of iv-FHSS. Section 4 investigates the distance measures of iv-FHSS. Section 5 presents the decision system of iv-FHSS along with application in the treatment of omicron patients. In the last section, the article is summarized with future directions.

## 2. Preliminaries

Let Z,P(Z),C(Z) represent the universe of discourse, collection of all subsets of Z and collection of all fuzzy sets of Z, respectively, and E be the set of parameters.

**Definition** **1**  ([3])**.** *A fuzzy set F over Z is characterized by a membership function $f_{F}:Z\;\to \;\left[0,1\right]$ and is given by $f_{F}\left(z\right)=\left\{(z,f_{F}\left(z\right))|z\in Z\right\}$ which assigns a real value within [0,1] to each z∈Z and $f_{F}\left(z\right)$ is the membership-grade of z∈Z.*

**Definition** **2**  ([4])**.** *A soft set $S_{S}$ over Z is defined as $\Lambda_{S_{S}}:E^{\prime }\to P\left(Z\right)$ where $E^{\prime }\subseteq E$ and is given by $S_{S}=\left\{\left(\theta ,\Lambda_{\left(S,E)(\theta \right)}\right):\theta \in E^{\prime }\right\}$.*

**Definition** **3**  ([5])**.** *A fuzzy soft set (fs-set) $F_{S_{S}}$ over Z is defined as $\Delta_{F_{S_{S}}}:E^{\prime }\to C\left(Z\right)$ and is given by $F_{S_{S}}=\left\{\left(\theta ,\Delta_{F_{S_{S}}}\left(\theta \right)\right):\theta \in E^{\prime }\right\},\Delta_{F_{S_{S}}}\left(\theta \right)\in C\left(Z\right)$ for $E^{\prime }\subseteq E$, $\Delta_{F_{S_{S}}}=\phi$ for $\theta \notin E^{\prime }$ and $\Delta_{F_{S_{E}^{\prime }}}\left(\theta \right)=\left\{\zeta_{\Delta_{F_{S_{E}^{\prime }}}\left(\theta \right)}\left(\omega \right)/\omega :\omega \in Z,\zeta_{\Delta_{F_{S_{E}^{\prime }}}\left(\theta \right)}\left(\omega \right)\in I\right\}$; for all $\theta \in E^{\prime }$ is an fs over Z, where $\Delta_{F_{S_{E}^{\prime }}}$ is the approximate function of $F_{S}$, and $\Delta_{F_{S}}\left(z\right)$ is a fuzzy set with the condition that if $\Delta_{F_{S_{E}^{\prime }}}\left(\theta \right)=\phi$, then $\left(\theta ,\Delta_{F_{S_{E}^{\prime }}}\left(\theta \right)\right)\notin F_{S_{E}^{\prime }}$.*

**Definition** **4**  ([7])**.** *An interval-valued fuzzy set (ivf-set) $M^{ivf}$ over Z is given by a function $I_{M^{ivf}}:Z\to I\left(\left[0,1\right]\right)$ where I([0,1]) is the set of all the sub-intervals of [0,1], $I_{M^{ivf}}\left(z\right)$ for all z∈Z is an interval [υ,ν],0≤υ≤ν≤1, and υ and ν denote lower and upper membership-grades of an element, respectively. For convenience, the set of all ivf-sets over Z is denoted by Γ(Z).*

**Definition** **5**  ([6])**.** *The collection of pairs $\left({\tilde{F}}_{S},E\right)$ is called interval-valued fuzzy soft set (ivfs-set) over Z and is given by ${\tilde{F}}_{S}:E\to P\left(Z\right)$. An ivfs-set is a parameterized family of ivf-subsets of Z and is defined as ${\tilde{F}}_{S}\left(\theta \right)=\left\{<\eta ,\kappa_{{\tilde{F}}_{S}\left(\theta \right)}\left(\eta \right)>:\eta \in Z,\theta \in E\right\}$ where ${\tilde{F}}_{S}\left(\theta \right)$ represent In-vf membership degree of η∈Z,θ∈E. ${\tilde{F}}_{S}\left(\theta \right)$ will become fuzzy set if $\kappa_{{\tilde{F}}_{S}\left(\theta \right)}^{-}\left(\eta \right)=\kappa_{{\tilde{F}}_{S}\left(\theta \right)}^{+}\left(\eta \right)$ for all η∈Z,θ∈E.*

**Example** **1.** *Let $Z=\left\{z_{1},z_{2},\ldots ,z_{6}\right\}$ be the set of six houses under consideration as in Example 1 discussed by [6]. Let $E=\left\{\theta_{1},\theta_{2},\ldots ,\theta_{5}\right\}=$ {cheap, beautiful, in green surroundings, wooden, good location} be the set of parameters where $\theta_{1}$ stands for cheapness, $\theta_{2}$ stands for beautiful appearance of house, $\theta_{3}$ stands for green surroundings of house, $\theta_{4}$ stands for material used to build house and $\theta_{5}$ stands for good location of house, i.e., near market, park, etc. Table 1 shows the tabular representation of ivfs-set.*

**Definition** **6**  ([17])**.** *$\left({\tilde{H}}_{S},E\right)$ is called hypersoft set (hs-set) over Z where $\epsilon_{1},\epsilon_{2},\ldots ,\epsilon_{n}$ are distinct attributes belonging to disjoint attribute valued sets $E_{1},E_{2},\ldots ,E_{n}$, respectively, and $E=E_{1}\times E_{2}\times \ldots \times E_{n}$ are the cartesian product of disjoint-attribute-valued sets and ${\tilde{H}}_{S}:E\to P\left(Z\right)$.*

**Example** **2.** *Consider Example 1. Let $Z=\left\{z_{1},z_{2},\ldots ,z_{6}\right\}$ be the set of six houses under consideration. Let $E=\left\{\epsilon_{1},\epsilon_{2},\ldots ,\epsilon_{5}\right\}=$ {cheap, beautiful, in green surroundings, wooden, good location} be the set of parameters where $\epsilon_{1}$ stands for cheap value, $\epsilon_{2}$ stands for beautiful appearance of house, $\epsilon_{3}$ stands for green surroundings of house, $\epsilon_{4}$ stands for material used to build house and $\epsilon_{5}$ stands for good location of house, i.e., near market, park, etc. The attribute valued sets corresponding to these attributes are: $E_{1}=\left\{\theta_{11},\theta_{12}\right\}$, $E_{2}=\left\{\theta_{21},\theta_{22}\right\}$, $E_{3}=\left\{\theta_{31}\right\}$, $E_{4}=\left\{\theta_{41}\right\}$ and $E_{5}=\left\{\theta_{51},\theta_{52}\right\}$. Then, $E=E_{1}\times E_{2}\times E_{3}\times E_{4}\times E_{5}$. $E=\left\{\upsilon_{1},\upsilon_{2},\ldots ,\upsilon_{8}\right\}$ where each $\upsilon_{i},i=1,2,\ldots ,8$, is a 5-tuple element. The hypersoft set $\left({\tilde{H}}_{S},E\right)$ is given as*

$$\left({\tilde{H}}_{S},E\right)=\left\{\begin{array}{c} \left(\upsilon_{1},\left\{z_{1},z_{2}\right\}\right),\left(\upsilon_{2},\left\{z_{1},z_{2},z_{3}\right\}\right),\left(\upsilon_{3},\left\{z_{1},z_{5},z_{6}\right\}\right),\left(\upsilon_{4},\left\{z_{2},z_{4},z_{5},z_{6}\right\}\right),\\ \left(\upsilon_{5},\left\{z_{1},z_{2},z_{6}\right\}\right),\left(\upsilon_{6},\left\{z_{3},z_{4},z_{5}\right\}\right),\left(\upsilon_{7},\left\{z_{1},z_{3},z_{4},z_{6}\right\}\right),\left(\upsilon_{8},\left\{z_{2},z_{3},z_{4},z_{5},z_{6}\right\}\right) \end{array}\right\}.$$

**Definition** **7**  ([17])**.** *Let $\epsilon_{1},\epsilon_{2},\ldots ,and\;\epsilon_{n}$ be distinct attributes belonging to disjoint-attribute-valued sets $E_{1},E_{2},\ldots ,E_{n}$ such that for $i,j=1,2,\ldots ,n,i\neq j,E_{i}\cap E_{j}=\phi$, fuzzy hypersoft set (fhs-set) $\left({\tilde{F}}_{HS},E\right)$ over Z is given by a set of ordered pairs*

*$\left({\tilde{F}}_{HS},E\right)=\left\{\left(\upsilon ,{\tilde{F}}_{HS}\left(\upsilon \right)\right):\upsilon \in E,{\tilde{F}}_{HS}\left(\upsilon \right)\in C\left(Z\right)\right\}$ where ${\tilde{F}}_{HS}:E\to C\left(Z\right)$ and C(Z) are the collection of all fuzzy sets over Z with $\upsilon \in E=E_{1}\times E_{2}\times \ldots \times E_{n}$, then ${\tilde{F}}_{HS}\left(\upsilon \right)=\left\{\kappa_{{\tilde{F}}_{HS}\left(\upsilon \right)}\left(z\right)/z:z\in Z,\kappa_{{\tilde{F}}_{HS}\left(\upsilon \right)}\in \left[0,1\right]\right\}$ is a fuzzy set over Z.*

**Example** **3.** *Consider Example 2. The fhs-set $\left({\tilde{F}}_{HS},E\right)$ is given as follows:*

$$\left({\tilde{F}}_{HS},E\right)=\left\{\begin{array}{c} \left(\upsilon_{1},\left\{0.1/z_{1},0.3/z_{2}\right\}\right),\left(\upsilon_{2},\left\{0.3/z_{1},0.5/z_{2},0.1/z_{3}\right\}\right),\\ \left(\upsilon_{3},\left\{0.2/z_{1},0.5/z_{5},0.3/z_{6}\right\}\right),\left(\upsilon_{4},\left\{0.2/z_{2},0.3/z_{4},0.4/z_{5},0.7/z_{6}\right\}\right),\\ \left(\upsilon_{5},\left\{0.3/z_{1},0.4/z_{2},0.5/z_{6}\right\}\right),\left(\upsilon_{6},\left\{0.1/z_{3},0.3/z_{4},0.2/z_{5}\right\}\right),\\ \left(\upsilon_{7},\left\{0.2/z_{1},0.3/z_{3},0.4/z_{4},0.5/z_{6}\right\}\right),\\ \left(\upsilon_{8},\left\{0.1/z_{2},0.2/z_{3},0.3/z_{4},0.4/z_{5},0.5/z_{6}\right\}\right) \end{array}\right\}.$$

**Definition** **8**  ([18])**.** *Let $\epsilon_{1},\epsilon_{2},\ldots ,\epsilon_{n}$ be distinct attributes belonging to disjoint-attribute-valued sets $E_{1},E_{2},\ldots ,E_{n}$ such that for $i,j=1,2,\ldots ,n,i\neq j,E_{i}\cap E_{j}=\phi$, then the interval-valued fuzzy hypersoft set (ivfhs-set) $\left({\tilde{F}}_{HS}^{ivf},E\right)$ over Z is given by the set of ordered pairs $\left({\tilde{F}}_{HS}^{ivf},E\right)=\left\{\left(\upsilon ,{\tilde{F}}_{HS}^{ivf}\left(\upsilon \right)\right):\upsilon \in E,{\tilde{F}}_{HS}^{ivf}\left(\upsilon \right)\in C^{ivf}\left(Z\right)\right\}$ where ${\tilde{F}}_{HS}^{ivf}:E\to C^{ivf}\left(Z\right)$ and $C^{ivf}\left(Z\right)$ are the collection of all ivf-sets over Z with $\upsilon \in E=E_{1}\times E_{2}\times \ldots \times E_{n}$, then, ${\tilde{F}}_{HS}^{ivf}\left(\upsilon \right)=\left\{\kappa_{{\tilde{F}}_{HS}^{ivf}\left(\upsilon \right)}\left(z\right)/z:z\in Z,\kappa_{{\tilde{F}}_{HS}^{ivf}\left(\upsilon \right)}\in \left[0,1\right]\right\}$ is ivf-set over Z.*

**Example** **4.** *Consider Example 3. The iv-FHSS $\left({\tilde{F}}_{HS}^{ivf},E\right)$ is given as follows: $({\tilde{F}}_{HS}^{ivf},E)=$*

$$\left\{\begin{array}{c} \left(\upsilon_{1},\left\{\left[0.1,0.2\right]/z_{1},\left[0.3,0.5\right]/z_{2}\right\}\right),\left(\upsilon_{2},\left\{\left[0.1,0.3\right]/z_{1},\left[0.2,0.4\right]/z_{2},\left[0.1,0.4\right]/z_{3}\right\}\right),\\ \left(\upsilon_{3},\left\{\left[0.2,0.6\right]/z_{1},\left[0.3,0.5\right]/z_{5},\left[0.2,0.3\right]/z_{6}\right\}\right),\\ \left(\upsilon_{4},\left\{\left[0.2,0.4\right]/z_{2},\left[0.5,0.7\right]/z_{4},\left[0.4,0.6\right]/z_{5},\left[0.5,0.7\right]/z_{6}\right\}\right),\\ \left(\upsilon_{5},\left\{\left[0.3,0.6\right]/z_{1},\left[0.4,0.7\right]/z_{2},\left[0.7,0.8\right]/z_{6}\right\}\right),\left(\upsilon_{6},\left\{\left[0.4,0.5\right]/z_{3},\left[0.3,0.5\right]/z_{4},\left[0.2,0.7\right]/z_{5}\right\}\right),\\ \left(\upsilon_{7},\left\{\left[0.2,0.5\right]/z_{1},\left[0.3,0.5\right]/z_{3},\left[0.4,0.6\right]/z_{4},\left[0.5,0.7\right]/z_{6}\right\}\right),\\ \left(\upsilon_{8},\left\{\left[0.1,0.5\right]/z_{2},\left[0.2,0.3\right]/z_{3},\left[0.1,0.3\right]/z_{4},\left[0.4,0.6\right]/z_{5},\left[0.3,0.5\right]/z_{6}\right\}\right) \end{array}\right\}.$$

## 3. Set Theoretic Operations of Interval-Valued Fuzzy Hypersoft Sets

This section of the paper aims to characterize some new operations of iv-FHSS. Consider two iv-FHSSs $\left(\tilde{F}{{}^{ivf}}_{HS}^{1},\Lambda_{1}\right)$ and $\left(\tilde{F}{{}^{ivf}}_{HS}^{2},\Lambda_{2}\right)$.

1. The addition of these two iv-FHSSs $\left(\tilde{F}{{}^{ivf}}_{HS}^{1},\Lambda_{1}\right)$ and $\left(\tilde{F}{{}^{ivf}}_{HS}^{2},\Lambda_{2}\right)$ is defined as follows: $\left(\tilde{F}{{}^{ivf}}_{HS}^{1},\Lambda_{1}\right)⊞\left(\tilde{F}{{}^{ivf}}_{HS}^{2},\Lambda_{2}\right)=\left\{\tilde{F}{{}^{ivf}}_{HS}^{1}\left(\eta \right)⊕\tilde{F}{{}^{ivf}}_{HS}^{2}\left(\eta \right):\eta \in \Lambda_{1}\cap \Lambda_{2}\right\}$ where
  $\tilde{F}{{}^{ivf}}_{HS}^{1}\left(\eta \right)⊕\tilde{F}{{}^{ivf}}_{HS}^{2}\left(\eta \right)=$
  $\left\{\left(z,\left[\begin{array}{c} \kappa_{l\tilde{F}{{}^{ivf}}_{HS}^{1}\left(\eta \right)}\left(z\right)+\kappa_{l\tilde{F}{{}^{ivf}}_{HS}^{2}\left(\eta \right)}\left(z\right)-\kappa_{l\tilde{F}{{}^{ivf}}_{HS}^{1}\left(\eta \right)}\left(z\right)\kappa_{l\tilde{F}{{}^{ivf}}_{HS}^{2}\left(\eta \right)}\left(z\right),\\ \kappa_{u\tilde{F}{{}^{ivf}}_{HS}^{1}\left(\eta \right)}\left(z\right)+\kappa_{u\tilde{F}{{}^{ivf}}_{HS}^{2}\left(\eta \right)}\left(z\right)-\kappa_{u\tilde{F}{{}^{ivf}}_{HS}^{1}\left(\eta \right)}\left(z\right)\kappa_{u\tilde{F}{{}^{ivf}}_{HS}^{2}\left(\eta \right)}\left(z\right) \end{array}\right]\right)\forall z\in Z\right\}$.
2. The multiplication of two iv-FHSSs $\left(\tilde{F}{{}^{ivf}}_{HS}^{1},\Lambda_{1}\right)$ and $\left(\tilde{F}{{}^{ivf}}_{HS}^{2},\Lambda_{2}\right)$ is defined as follow:
  $\left(\tilde{F}{{}^{ivf}}_{HS}^{1},\Lambda_{1}\right)⊠\left(\tilde{F}{{}^{ivf}}_{HS}^{2},\Lambda_{2}\right)=\left\{\tilde{F}{{}^{ivf}}_{HS}^{1}\left(\eta \right)⊗\tilde{F}{{}^{ivf}}_{HS}^{2}\left(\eta \right):\eta \in \Lambda_{1}\cap \Lambda_{2}\right\}$ where $\tilde{F}{{}^{ivf}}_{HS}^{1}\left(\eta \right)⊗\tilde{F}{{}^{ivf}}_{HS}^{2}\left(\eta \right)=\left\{\left(z,\left[\min\left\{\kappa_{l\tilde{F}{{}^{ivf}}_{HS}^{1}\left(\eta \right)}\left(z\right),\kappa_{l\tilde{F}{{}^{ivf}}_{HS}^{2}\left(\eta \right)}\left(z\right)\right\}\right.\right.\right.$, $\left.\left.\left.\max\left\{\kappa_{u\tilde{F}{{}^{ivf}}_{HS}^{1}\left(\eta \right)}\left(z\right),\kappa_{u\tilde{F}{{}^{ivf}}_{HS}^{2}\left(\eta \right)}\left(z\right)\right\}\right]\right)\forall z\in Z\right\}$.
3. The union of two iv-FHSSs $\left(\tilde{F}{{}^{ivf}}_{HS}^{1},\Lambda_{1}\right)$ and $\left(\tilde{F}{{}^{ivf}}_{HS}^{2},\Lambda_{2}\right)$ is defined as follows: $\left(\tilde{F}{{}^{ivf}}_{HS}^{1},\Lambda_{1}\right)⊔\left(\tilde{F}{{}^{ivf}}_{HS}^{2},\Lambda_{2}\right)=\left\{\tilde{F}{{}^{ivf}}_{HS}^{1}\left(\eta \right)\cup \tilde{F}{{}^{ivf}}_{HS}^{2}\left(\eta \right):\eta \in \Lambda_{1}\cap \Lambda_{2}\right\}$ where $\tilde{F}{{}^{ivf}}_{HS}^{1}\left(\eta \right)\cup \tilde{F}{{}^{ivf}}_{HS}^{2}\left(\eta \right)=\left\{\left(z,\left[\max\left(\kappa_{l\tilde{F}{{}^{ivf}}_{HS}^{1}\left(\eta \right)}\left(z\right),\kappa_{l\tilde{F}{{}^{ivf}}_{HS}^{2}\left(\eta \right)}\left(z\right)\right)\right.\right.\right.$, $\left.\left.\left.\max\left(\kappa_{u\tilde{F}{{}^{ivf}}_{HS}^{1}\left(\eta \right)}\left(z\right),\kappa_{u\tilde{F}{{}^{ivf}}_{HS}^{2}\left(\eta \right)}\left(z\right)\right)\right]\right)\forall z\in Z\right\}$.
4. The intersection of two iv-FHSSs $\left(\tilde{F}{{}^{ivf}}_{HS}^{1},\Lambda_{1}\right)$ and $\left(\tilde{F}{{}^{ivf}}_{HS}^{2},\Lambda_{2}\right)$ is defined as follows: $\left(\tilde{F}{{}^{ivf}}_{HS}^{1},\Lambda_{1}\right)⊓\left(\tilde{F}{{}^{ivf}}_{HS}^{2},\Lambda_{2}\right)=\left\{\tilde{F}{{}^{ivf}}_{HS}^{1}\left(\eta \right)\cap \tilde{F}{{}^{ivf}}_{HS}^{2}\left(\eta \right):\eta \in \Lambda_{1}\cap \Lambda_{2}\right\}$ where $\tilde{F}{{}^{ivf}}_{HS}^{1}\left(\eta \right)\cap \tilde{F}{{}^{ivf}}_{HS}^{2}\left(\eta \right)=\left\{\left(z,\left[\min\left(\kappa_{l\tilde{F}{{}^{ivf}}_{HS}^{1}\left(\eta \right)}\left(z\right),\kappa_{l\tilde{F}{{}^{ivf}}_{HS}^{2}\left(\eta \right)}\left(z\right)\right)\right.\right.\right.$, $\left.\left.\left.\min\left(\kappa_{u\tilde{F}{{}^{ivf}}_{HS}^{1}\left(\eta \right)}\left(z\right),\kappa_{u\tilde{F}{{}^{ivf}}_{HS}^{2}\left(\eta \right)}\left(z\right)\right)\right]\right)\forall z\in Z\right\}$.
5. Partial membership of iv-FHSS $\left(\tilde{F}{{}^{ivf}}_{HS}^{1},\Lambda_{1}\right)$ is defined as follows: $⊡\left(\tilde{F}{{}^{ivf}}_{HS}^{1},\Lambda_{1}\right)=\left\{⊙\tilde{F}{{}^{ivf}}_{HS}^{1}\left(\eta \right):\eta \in \Lambda_{1}\right\}$ where $⊙\tilde{F}{{}^{ivf}}_{HS}^{1}\left(\eta \right)=\left\{z,\left[\kappa_{l\tilde{F}{{}^{ivf}}_{HS}^{1}\left(\eta \right)}\left(z\right),1-\kappa_{l\tilde{F}{{}^{ivf}}_{HS}^{1}\left(\eta \right)}\left(z\right)\right]\forall z\in Z\right\}$.
6. Partial non-membership of iv-FHSS $\left(\tilde{F}{{}^{ivf}}_{HS}^{1},\Lambda_{1}\right)$ is defined as follows: $⊚\left(\tilde{F}{{}^{ivf}}_{HS}^{1},\Lambda_{1}\right)=\left\{\circ \tilde{F}{{}^{ivf}}_{HS}^{1}\left(\eta \right):\eta \in \Lambda_{1}\cap \Lambda_{2}\right\}$ where $\circ \tilde{F}{{}^{ivf}}_{HS}^{1}\left(\eta \right)=\left\{z,\left[1-\kappa_{u\tilde{F}{{}^{ivf}}_{HS}^{1}\left(\eta \right)}\left(z\right),\kappa_{u\tilde{F}{{}^{ivf}}_{HS}^{1}\left(\eta \right)}\left(z\right)\right]\forall z\in Z\right\}$.

**Example** **5.** *An application of a similarity measure on fuzzy soft sets in medical diagnosis is presented in [35]. In this section, an application on iv-FHSS is discussed. Let a businessman want to hire a contractor for the construction of a building. There are five contractors $Z=\left\{z_{1},z_{2},\ldots ,z_{5}\right\}$ under consideration. There are two committees of experts. One of the committee considers a set of attributes $E=\left\{\Omega_{1},\Omega_{2},\Omega_{3}\right\}$ where $\Omega_{1}=$ Availability of experienced staff $=\left\{\leq 25,>25\right\}=\left\{\omega_{11},\omega_{12}\right\}$, $\Omega_{2}=$ Number of projects completed on time $=\left\{\leq 5,>5\right\}=\left\{\omega_{21},\omega_{22}\right\}$, $\Omega_{3}=$ Availability of heavy machinery = {available, not available} $=\left\{\omega_{31},\omega_{32}\right\}$, and $\Omega =\Omega_{1}\times \Omega_{2}\times \Omega_{3}$*

$$=\left\{\begin{array}{c} \left(\omega_{11},\omega_{21},\omega_{31}\right),\left(\omega_{11},\omega_{21},\omega_{32}\right),\left(\omega_{11},\omega_{22},\omega_{31}\right),\left(\omega_{11},\omega_{22},\omega_{32}\right),\\ \left(\omega_{12},\omega_{21},\omega_{31}\right),\left(\omega_{12},\omega_{21},\omega_{32}\right),\left(\omega_{12},\omega_{22},\omega_{31}\right),\left(\omega_{12},\omega_{22},\omega_{32}\right) \end{array}\right\}=\left\{\omega_{1},\omega_{2},\ldots ,\omega_{8}\right\}.$$

*The iv-FHSS $\tilde{F}{{}^{ivf}}_{HS}^{1}\left(\Omega \right)=\left\{\tilde{F}{{}^{ivf}}_{HS}^{1}\left(\omega_{1}\right),\tilde{F}{{}^{ivf}}_{HS}^{1}\left(\omega_{2}\right),\ldots ,\tilde{F}{{}^{ivf}}_{HS}^{1}\left(\omega_{8}\right)\right\}$ where*

$$\tilde{F}{{}^{ivf}}_{HS}^{1}\left(\omega_{1}\right)=\left\{\frac{\left[0.6,0.7\right]}{z_{1}},\frac{\left[0.2,0.3\right]}{z_{2}},\frac{\left[0.5,0.6\right]}{z_{3}},\frac{\left[0.4,0.5\right]}{z_{4}},\frac{\left[0.3,0.4\right]}{z_{5}}\right\},$$

$$\tilde{F}{{}^{ivf}}_{HS}^{1}\left(\omega_{2}\right)=\left\{\frac{\left[0.3,0.5\right]}{z_{1}},\frac{\left[0.2,0.4\right]}{z_{2}},\frac{\left[0.2,0.8\right]}{z_{3}},\frac{\left[0.4,0.5\right]}{z_{4}},\frac{\left[0.2,0.6\right]}{z_{5}}\right\},$$

$$\tilde{F}{{}^{ivf}}_{HS}^{1}\left(\omega_{3}\right)=\left\{\frac{\left[0.3,0.5\right]}{z_{1}},\frac{\left[0.1,0.4\right]}{z_{2}},\frac{\left[0.3,0.6\right]}{z_{3}},\frac{\left[0.2,0.6\right]}{z_{4}},\frac{\left[0.3,0.5\right]}{z_{5}}\right\},$$

$$\tilde{F}{{}^{ivf}}_{HS}^{1}\left(\omega_{4}\right)=\left\{\frac{\left[0.2,0.6\right]}{z_{1}},\frac{\left[0.4,0.6\right]}{z_{2}},\frac{\left[0.2,0.4\right]}{z_{3}},\frac{\left[0.3,0.7\right]}{z_{4}},\frac{\left[0.1,0.5\right]}{z_{5}}\right\},$$

$$\tilde{F}{{}^{ivf}}_{HS}^{1}\left(\omega_{5}\right)=\left\{\frac{\left[0.3,0.5\right]}{z_{1}},\frac{\left[0.2,0.6\right]}{z_{2}},\frac{\left[0.1,0.4\right]}{z_{3}},\frac{\left[0.2,0.8\right]}{z_{4}},\frac{\left[0.1,0.4\right]}{z_{5}}\right\},$$

$$\tilde{F}{{}^{ivf}}_{HS}^{1}\left(\omega_{6}\right)=\left\{\frac{\left[0.4,0.5\right]}{z_{1}},\frac{\left[0.1,0.3\right]}{z_{2}},\frac{\left[0.4,0.7\right]}{z_{3}},\frac{\left[0.2,0.5\right]}{z_{4}},\frac{\left[0.3,0.7\right]}{z_{5}}\right\},$$

$$\tilde{F}{{}^{ivf}}_{HS}^{1}\left(\omega_{7}\right)=\left\{\frac{\left[0.1,0.4\right]}{z_{1}},\frac{\left[0.1,0.5\right]}{z_{2}},\frac{\left[0.2,0.6\right]}{z_{3}},\frac{\left[0.4,0.5\right]}{z_{4}},\frac{\left[0.3,0.4\right]}{z_{5}}\right\},$$

$$\tilde{F}{{}^{ivf}}_{HS}^{1}\left(\omega_{8}\right)=\left\{\frac{\left[0.5,0.9\right]}{z_{1}},\frac{\left[0.2,0.5\right]}{z_{2}},\frac{\left[0.3,0.7\right]}{z_{3}},\frac{\left[0.2,0.5\right]}{z_{4}},\frac{\left[0.1,0.4\right]}{z_{5}}\right\}.$$

*The other committee constructed iv-FHSS* *$\tilde{F}{{}^{ivf}}_{HS}^{2}\left(\omega \right)=\left\{\tilde{F}{{}^{ivf}}_{HS}^{2}\left(\omega_{1}\right),\tilde{F}{{}^{ivf}}_{HS}^{2}\left(\omega_{2}\right),\ldots ,\tilde{F}{{}^{ivf}}_{HS}^{2}\left(\omega_{8}\right)\right\}$ where*

$$\tilde{F}{{}^{ivf}}_{HS}^{2}\left(\omega_{1}\right)=\left\{\frac{\left[0.3,0.4\right]}{z_{1}},\frac{\left[0.3,0.6\right]}{z_{2}},\frac{\left[0.3,0.5\right]}{z_{3}},\frac{\left[0.2,0.5\right]}{z_{4}},\frac{\left[0.3,0.6\right]}{z_{5}}\right\},$$

$$\tilde{F}{{}^{ivf}}_{HS}^{2}\left(\omega_{2}\right)=\left\{\frac{\left[0.4,0.6\right]}{z_{1}},\frac{\left[0.2,0.5\right]}{z_{2}},\frac{\left[0.4,0.7\right]}{z_{3}},\frac{\left[0.3,0.5\right]}{z_{4}},\frac{\left[0.5,0.7\right]}{z_{5}}\right\},$$

$$\tilde{F}{{}^{ivf}}_{HS}^{2}\left(\omega_{3}\right)=\left\{\frac{\left[0.4,0.6\right]}{z_{1}},\frac{\left[0.1,0.5\right]}{z_{2}},\frac{\left[0.5,0.7\right]}{z_{3}},\frac{\left[0.4,0.6\right]}{z_{4}},\frac{\left[0.3,0.6\right]}{z_{5}}\right\},$$

$$\tilde{F}{{}^{ivf}}_{HS}^{2}\left(\omega_{4}\right)=\left\{\frac{\left[0.2,0.6\right]}{z_{1}},\frac{\left[0.4,0.7\right]}{z_{2}},\frac{\left[0.1,0.3\right]}{z_{3}},\frac{\left[0.4,0.8\right]}{z_{4}},\frac{\left[0.2,0.5\right]}{z_{5}}\right\},$$

$$\tilde{F}{{}^{ivf}}_{HS}^{2}\left(\omega_{5}\right)=\left\{\frac{\left[0.4,0.5\right]}{z_{1}},\frac{\left[0.3,0.6\right]}{z_{2}},\frac{\left[0.3,0.4\right]}{z_{3}},\frac{\left[0.4,0.6\right]}{z_{4}},\frac{\left[0.3,0.5\right]}{z_{5}}\right\},$$

$$\tilde{F}{{}^{ivf}}_{HS}^{2}\left(\omega_{6}\right)=\left\{\frac{\left[0.4,0.5\right]}{z_{1}},\frac{\left[0.3,0.5\right]}{z_{2}},\frac{\left[0.5,0.6\right]}{z_{3}},\frac{\left[0.3,0.6\right]}{z_{4}},\frac{\left[0.4,0.8\right]}{z_{5}}\right\},$$

$$\tilde{F}{{}^{ivf}}_{HS}^{2}\left(\omega_{7}\right)=\left\{\frac{\left[0.3,0.5\right]}{z_{1}},\frac{\left[0.2,0.6\right]}{z_{2}},\frac{\left[0.3,0.6\right]}{z_{3}},\frac{\left[0.2,0.4\right]}{z_{4}},\frac{\left[0.5,0.7\right]}{z_{5}}\right\},$$

$$\tilde{F}{{}^{ivf}}_{HS}^{2}\left(\omega_{8}\right)=\left\{\frac{\left[0.7,0.9\right]}{z_{1}},\frac{\left[0.4,0.6\right]}{z_{2}},\frac{\left[0.4,0.8\right]}{z_{3}},\frac{\left[0.3,0.5\right]}{z_{4}},\frac{\left[0.3,0.6\right]}{z_{5}}\right\}.$$

*The iv-FHSS $\left(\tilde{F}{{}^{ivf}}_{HS}^{1},\Omega \right)$ can also be written as $(\tilde{F}{{}^{ivf}}_{HS}^{1},\Omega )=$*

$$\left\{\begin{array}{c} \left(\omega_{1},\frac{\left[0.6,0.7\right]}{z_{1}},\frac{\left[0.2,0.3\right]}{z_{2}},\frac{\left[0.5,0.6\right]}{z_{3}},\frac{\left[0.4,0.5\right]}{z_{4}},\frac{\left[0.3,0.4\right]}{z_{5}}\right),\left(\omega_{2},\frac{\left[0.3,0.5\right]}{z_{1}},\frac{\left[0.2,0.4\right]}{z_{2}},\frac{\left[0.2,0.8\right]}{z_{3}},\frac{\left[0.4,0.5\right]}{z_{4}},\frac{\left[0.2,0.6\right]}{z_{5}}\right)\\ \left(\omega_{3},\frac{\left[0.3,0.5\right]}{z_{1}},\frac{\left[0.1,0.4\right]}{z_{2}},\frac{\left[0.3,0.6\right]}{z_{3}},\frac{\left[0.2,0.6\right]}{z_{4}},\frac{\left[0.3,0.5\right]}{z_{5}}\right),\left(\omega_{4},\frac{\left[0.2,0.6\right]}{z_{1}},\frac{\left[0.4,0.6\right]}{z_{2}},\frac{\left[0.2,0.4\right]}{z_{3}},\frac{\left[0.3,0.7\right]}{z_{4}},\frac{\left[0.1,0.5\right]}{z_{5}}\right)\\ \left(\omega_{5},\frac{\left[0.3,0.5\right]}{z_{1}},\frac{\left[0.2,0.6\right]}{z_{2}},\frac{\left[0.1,0.4\right]}{z_{3}},\frac{\left[0.2,0.8\right]}{z_{4}},\frac{\left[0.1,0.4\right]}{z_{5}}\right),\left(\omega_{6},\frac{\left[0.4,0.5\right]}{z_{1}},\frac{\left[0.1,0.3\right]}{z_{2}},\frac{\left[0.4,0.7\right]}{z_{3}},\frac{\left[0.2,0.5\right]}{z_{4}},\frac{\left[0.3,0.7\right]}{z_{5}}\right)\\ \left(\omega_{7},\frac{\left[0.1,0.4\right]}{z_{1}},\frac{\left[0.1,0.5\right]}{z_{2}},\frac{\left[0.2,0.6\right]}{z_{3}},\frac{\left[0.4,0.5\right]}{z_{4}},\frac{\left[0.3,0.4\right]}{z_{5}}\right),\left(\omega_{8},\frac{\left[0.5,0.9\right]}{z_{1}},\frac{\left[0.2,0.5\right]}{z_{2}},\frac{\left[0.3,0.7\right]}{z_{3}},\frac{\left[0.2,0.5\right]}{z_{4}},\frac{\left[0.1,0.4\right]}{z_{5}}\right) \end{array}\right\}.$$

*Similarly, iv-FHSS $\left(\tilde{F}{{}^{ivf}}_{HS}^{2},\Omega \right)$ can also be written as $(\tilde{F}{{}^{ivf}}_{HS}^{2},\Omega )=$*

$$\left\{\begin{array}{c} \left(\omega_{1},\frac{\left[0.3,0.4\right]}{z_{1}},\frac{\left[0.3,0.6\right]}{z_{2}},\frac{\left[0.3,0.5\right]}{z_{3}},\frac{\left[0.2,0.5\right]}{z_{4}},\frac{\left[0.3,0.6\right]}{z_{5}}\right),\left(\omega_{2},\frac{\left[0.4,0.6\right]}{z_{1}},\frac{\left[0.2,0.5\right]}{z_{2}},\frac{\left[0.4,0.7\right]}{z_{3}},\frac{\left[0.3,0.5\right]}{z_{4}},\frac{\left[0.5,0.7\right]}{z_{5}}\right)\\ \left(\omega_{3},\frac{\left[0.4,0.6\right]}{z_{1}},\frac{\left[0.1,0.5\right]}{z_{2}},\frac{\left[0.5,0.7\right]}{z_{3}},\frac{\left[0.4,0.6\right]}{z_{4}},\frac{\left[0.3,0.6\right]}{z_{5}}\right),\left(\omega_{4},\frac{\left[0.2,0.6\right]}{z_{1}},\frac{\left[0.4,0.7\right]}{z_{2}},\frac{\left[0.1,0.3\right]}{z_{3}},\frac{\left[0.4,0.8\right]}{z_{4}},\frac{\left[0.2,0.5\right]}{z_{5}}\right)\\ \left(\omega_{5},\frac{\left[0.4,0.5\right]}{z_{1}},\frac{\left[0.3,0.6\right]}{z_{2}},\frac{\left[0.3,0.4\right]}{z_{3}},\frac{\left[0.4,0.6\right]}{z_{4}},\frac{\left[0.3,0.5\right]}{z_{5}}\right),\left(\omega_{6},\frac{\left[0.4,0.5\right]}{z_{1}},\frac{\left[0.3,0.5\right]}{z_{2}},\frac{\left[0.5,0.6\right]}{z_{3}},\frac{\left[0.3,0.6\right]}{z_{4}},\frac{\left[0.4,0.8\right]}{z_{5}}\right)\\ \left(\omega_{7},\frac{\left[0.3,0.5\right]}{z_{1}},\frac{\left[0.2,0.6\right]}{z_{2}},\frac{\left[0.3,0.6\right]}{z_{3}},\frac{\left[0.2,0.4\right]}{z_{4}},\frac{\left[0.5,0.7\right]}{z_{5}}\right),\left(\omega_{8},\frac{\left[0.7,0.9\right]}{z_{1}},\frac{\left[0.4,0.6\right]}{z_{2}},\frac{\left[0.4,0.8\right]}{z_{3}},\frac{\left[0.3,0.5\right]}{z_{4}},\frac{\left[0.3,0.6\right]}{z_{5}}\right) \end{array}\right\}.$$

**Example** **6.** *Consider two iv-FHSS $\left(\tilde{F}{{}^{ivf}}_{HS}^{1},\Omega \right)$ and $\left(\tilde{F}{{}^{ivf}}_{HS}^{2},\Omega \right)$ as in Example 5. The addition of $\left(\tilde{F}{{}^{ivf}}_{HS}^{1},\Omega \right)$ and $\left(\tilde{F}{{}^{ivf}}_{HS}^{2},\Omega \right)$ is calculated as follows:*

$$\left(\tilde{F}{{}^{ivf}}_{HS}^{1},\Omega \right)⊞\left(\tilde{F}{{}^{ivf}}_{HS}^{2},\Omega \right)=\left\{\begin{array}{c} \left(\omega_{1},\frac{\left[0.72,0.82\right]}{z_{1}},\frac{\left[0.44,0.72\right]}{z_{2}},\frac{\left[0.65,0.80\right]}{z_{3}},\frac{\left[0.52,0.75\right]}{z_{4}},\frac{\left[0.51,0.76\right]}{z_{5}}\right),\\ \left(\omega_{2},\frac{\left[0.58,0.80\right]}{z_{1}},\frac{\left[0.36,0.70\right]}{z_{2}},\frac{\left[0.52,0.94\right]}{z_{3}},\frac{\left[0.58,0.75\right]}{z_{4}},\frac{\left[0.60,0.88\right]}{z_{5}}\right)\\ \left(\omega_{3},\frac{\left[0.58,0.80\right]}{z_{1}},\frac{\left[0.19,0.70\right]}{z_{2}},\frac{\left[0.65,0.88\right]}{z_{3}},\frac{\left[0.36,0.84\right]}{z_{4}},\frac{\left[0.51,0.80\right]}{z_{5}}\right),\\ \left(\omega_{4},\frac{\left[0.36,0.84\right]}{z_{1}},\frac{\left[0.64,0.88\right]}{z_{2}},\frac{\left[0.28,0.58\right]}{z_{3}},\frac{\left[0.58,0.94\right]}{z_{4}},\frac{\left[0.28,0.75\right]}{z_{5}}\right)\\ \left(\omega_{5},\frac{\left[0.58,0.75\right]}{z_{1}},\frac{\left[0.44,0.84\right]}{z_{2}},\frac{\left[0.37,0.64\right]}{z_{3}},\frac{\left[0.52,0.92\right]}{z_{4}},\frac{\left[0.37,0.70\right]}{z_{5}}\right),\\ \left(\omega_{6},\frac{\left[0.64,0.75\right]}{z_{1}},\frac{\left[0.37,0.65\right]}{z_{2}},\frac{\left[0.70,0.88\right]}{z_{3}},\frac{\left[0.44,0.80\right]}{z_{4}},\frac{\left[0.58,0.94\right]}{z_{5}}\right)\\ \left(\omega_{7},\frac{\left[0.37,0.70\right]}{z_{1}},\frac{\left[0.28,0.80\right]}{z_{2}},\frac{\left[0.44,0.84\right]}{z_{3}},\frac{\left[0.52,0.70\right]}{z_{4}},\frac{\left[0.65,0.82\right]}{z_{5}}\right),\\ \left(\omega_{8},\frac{\left[0.85,0.99\right]}{z_{1}},\frac{\left[0.52,0.80\right]}{z_{2}},\frac{\left[0.58,0.94\right]}{z_{3}},\frac{\left[0.44,0.75\right]}{z_{4}},\frac{\left[0.37,0.76\right]}{z_{5}}\right) \end{array}\right\},\;\forall z\in Z.$$

**Example** **7.** *Consider two iv-FHSS $\left(\tilde{F}{{}^{ivf}}_{HS}^{1},\Omega \right)$ and $\left(\tilde{F}{{}^{ivf}}_{HS}^{2},\Omega \right)$ as in Example 5. The multiplication of iv-FHSSs $\left(\tilde{F}{{}^{ivf}}_{HS}^{1},\Omega \right)$ and $\left(\tilde{F}{{}^{ivf}}_{HS}^{2},\Omega^{{}^{\prime }}\right)$ is calculated as follows:* $\left(\tilde{F}{{}^{ivf}}_{HS}^{1},\Omega_{1}\right)⊠\left(\tilde{F}{{}^{ivf}}_{HS}^{2},\Omega_{2}\right)=\left\{\begin{array}{c} \left(\omega_{1},\frac{\left[0.3,0.7\right]}{z_{1}},\frac{\left[0.2,0.6\right]}{z_{2}},\frac{\left[0.3,0.6\right]}{z_{3}},\frac{\left[0.2,0.5\right]}{z_{4}},\frac{\left[0.3,0.6\right]}{z_{5}}\right),\left(\omega_{2},\frac{\left[0.3,0.6\right]}{z_{1}},\frac{\left[0.2,0.5\right]}{z_{2}},\frac{\left[0.2,0.8\right]}{z_{3}},\frac{\left[0.3,0.5\right]}{z_{4}},\frac{\left[0.2,0.7\right]}{z_{5}}\right)\\ \left(\omega_{3},\frac{\left[0.3,0.6\right]}{z_{1}},\frac{\left[0.1,0.5\right]}{z_{2}},\frac{\left[0.3,0.7\right]}{z_{3}},\frac{\left[0.2,0.6\right]}{z_{4}},\frac{\left[0.3,0.6\right]}{z_{5}}\right),\left(\omega_{4},\frac{\left[0.2,0.6\right]}{z_{1}},\frac{\left[0.4,0.7\right]}{z_{2}},\frac{\left[0.1,0.4\right]}{z_{3}},\frac{\left[0.3,0.8\right]}{z_{4}},\frac{\left[0.1,0.5\right]}{z_{5}}\right)\\ \left(\omega_{5},\frac{\left[0.3,0.5\right]}{z_{1}},\frac{\left[0.2,0.6\right]}{z_{2}},\frac{\left[0.1,0.4\right]}{z_{3}},\frac{\left[0.2,0.8\right]}{z_{4}},\frac{\left[0.1,0.5\right]}{z_{5}}\right),\left(\omega_{6},\frac{\left[0.4,0.5\right]}{z_{1}},\frac{\left[0.1,0.5\right]}{z_{2}},\frac{\left[0.4,0.7\right]}{z_{3}},\frac{\left[0.2,0.6\right]}{z_{4}},\frac{\left[0.3,0.8\right]}{z_{5}}\right)\\ \left(\omega_{7},\frac{\left[0.1,0.5\right]}{z_{1}},\frac{\left[0.1,0.6\right]}{z_{2}},\frac{\left[0.2,0.6\right]}{z_{3}},\frac{\left[0.2,0.5\right]}{z_{4}},\frac{\left[0.3,0.7\right]}{z_{5}}\right),\left(\omega_{8},\frac{\left[0.5,0.9\right]}{z_{1}},\frac{\left[0.2,0.6\right]}{z_{2}},\frac{\left[0.3,0.8\right]}{z_{3}},\frac{\left[0.2,0.5\right]}{z_{4}},\frac{\left[0.1,0.6\right]}{z_{5}}\right) \end{array}\right\},$ ∀z∈Z.

**Example** **8.** *Consider two iv-FHSS $\left({{\tilde{F}}^{ivf}}_{HS}^{1},\Omega \right)$ and $\left({{\tilde{F}}^{ivf}}_{HS}^{2},\Omega \right)$ as in Example 5. The union of iv-FHSSs $\left({{\tilde{F}}^{ivf}}_{HS}^{1},\Omega \right)$ and $\left({{\tilde{F}}^{ivf}}_{HS}^{2},\Omega \right)$ is calculated as follows:* $\left({{\tilde{F}}^{ivf}}_{HS}^{1},\Omega \right)⊔\left({{\tilde{F}}^{ivf}}_{HS}^{2},\Omega \right)=\left\{\begin{array}{c} \left(\omega_{1},\frac{\left[0.6,0.7\right]}{z_{1}},\frac{\left[0.3,0.6\right]}{z_{2}},\frac{\left[0.5,0.6\right]}{z_{3}},\frac{\left[0.4,0.5\right]}{z_{4}},\frac{\left[0.3,0.6\right]}{z_{5}}\right),\left(\omega_{2},\frac{\left[0.4,0.6\right]}{z_{1}},\frac{\left[0.2,0.5\right]}{z_{2}},\frac{\left[0.4,0.8\right]}{z_{3}},\frac{\left[0.4,0.5\right]}{z_{4}},\frac{\left[0.5,0.7\right]}{z_{5}}\right)\\ \left(\omega_{3},\frac{\left[0.4,0.6\right]}{z_{1}},\frac{\left[0.1,0.5\right]}{z_{2}},\frac{\left[0.5,0.7\right]}{z_{3}},\frac{\left[0.4,0.6\right]}{z_{4}},\frac{\left[0.3,0.6\right]}{z_{5}}\right),\left(\omega_{4},\frac{\left[0.2,0.6\right]}{z_{1}},\frac{\left[0.4,0.7\right]}{z_{2}},\frac{\left[0.2,0.4\right]}{z_{3}},\frac{\left[0.4,0.8\right]}{z_{4}},\frac{\left[0.2,0.5\right]}{z_{5}}\right)\\ \left(\omega_{5},\frac{\left[0.4,0.5\right]}{z_{1}},\frac{\left[0.3,0.6\right]}{z_{2}},\frac{\left[0.3,0.4\right]}{z_{3}},\frac{\left[0.4,0.8\right]}{z_{4}},\frac{\left[0.3,0.5\right]}{z_{5}}\right),\left(\omega_{6},\frac{\left[0.4,0.5\right]}{z_{1}},\frac{\left[0.3,0.5\right]}{z_{2}},\frac{\left[0.5,0.7\right]}{z_{3}},\frac{\left[0.3,0.6\right]}{z_{4}},\frac{\left[0.4,0.8\right]}{z_{5}}\right)\\ \left(\omega_{7},\frac{\left[0.3,0.5\right]}{z_{1}},\frac{\left[0.2,0.6\right]}{z_{2}},\frac{\left[0.3,0.6\right]}{z_{3}},\frac{\left[0.4,0.5\right]}{z_{4}},\frac{\left[0.5,0.7\right]}{z_{5}}\right),\left(\omega_{8},\frac{\left[0.7,0.9\right]}{z_{1}},\frac{\left[0.4,0.6\right]}{z_{2}},\frac{\left[0.4,0.8\right]}{z_{3}},\frac{\left[0.3,0.5\right]}{z_{4}},\frac{\left[0.3,0.6\right]}{z_{5}}\right) \end{array}\right\},$ ∀z∈Z.

**Example** **9.** *Consider two iv-FHSS $\left({{\tilde{F}}^{ivf}}_{HS}^{1},\Omega \right)$ and $\left({{\tilde{F}}^{ivf}}_{HS}^{2},\Omega \right)$ as in Example 5. The intersection of iv-FHSSs $\left({{\tilde{F}}^{ivf}}_{HS}^{1},\Omega \right)$ and $\left({{\tilde{F}}^{ivf}}_{HS}^{2},\Omega \right)$ is calculated as follows:* $\left({{\tilde{F}}^{ivf}}_{HS}^{1},\Omega_{1}\right)⊓\left({{\tilde{F}}^{ivf}}_{HS}^{2},\Omega_{2}\right)=\left\{\begin{array}{c} \left(\omega_{1},\frac{\left[0.3,0.4\right]}{z_{1}},\frac{\left[0.2,0.3\right]}{z_{2}},\frac{\left[0.3,0.5\right]}{z_{3}},\frac{\left[0.2,0.5\right]}{z_{4}},\frac{\left[0.3,0.4\right]}{z_{5}}\right),\left(\omega_{2},\frac{\left[0.3,0.5\right]}{z_{1}},\frac{\left[0.2,0.4\right]}{z_{2}},\frac{\left[0.2,0.7\right]}{z_{3}},\frac{\left[0.3,0.5\right]}{z_{4}},\frac{\left[0.2,0.6\right]}{z_{5}}\right)\\ \left(\omega_{3},\frac{\left[0.3,0.5\right]}{z_{1}},\frac{\left[0.1,0.4\right]}{z_{2}},\frac{\left[0.3,0.6\right]}{z_{3}},\frac{\left[0.2,0.6\right]}{z_{4}},\frac{\left[0.3,0.5\right]}{z_{5}}\right),\left(\omega_{4},\frac{\left[0.2,0.6\right]}{z_{1}},\frac{\left[0.4,0.6\right]}{z_{2}},\frac{\left[0.1,0.3\right]}{z_{3}},\frac{\left[0.3,0.7\right]}{z_{4}},\frac{\left[0.1,0.5\right]}{z_{5}}\right)\\ \left(\omega_{5},\frac{\left[0.3,0.5\right]}{z_{1}},\frac{\left[0.2,0.6\right]}{z_{2}},\frac{\left[0.1,0.4\right]}{z_{3}},\frac{\left[0.2,0.6\right]}{z_{4}},\frac{\left[0.1,0.4\right]}{z_{5}}\right),\left(\omega_{6},\frac{\left[0.4,0.5\right]}{z_{1}},\frac{\left[0.1,0.3\right]}{z_{2}},\frac{\left[0.4,0.6\right]}{z_{3}},\frac{\left[0.2,0.5\right]}{z_{4}},\frac{\left[0.3,0.7\right]}{z_{5}}\right)\\ \left(\omega_{7},\frac{\left[0.1,0.4\right]}{z_{1}},\frac{\left[0.1,0.5\right]}{z_{2}},\frac{\left[0.2,0.6\right]}{z_{3}},\frac{\left[0.2,0.4\right]}{z_{4}},\frac{\left[0.3,0.4\right]}{z_{5}}\right),\left(\omega_{8},\frac{\left[0.5,0.9\right]}{z_{1}},\frac{\left[0.2,0.5\right]}{z_{2}},\frac{\left[0.3,0.7\right]}{z_{3}},\frac{\left[0.2,0.5\right]}{z_{4}},\frac{\left[0.1,0.4\right]}{z_{5}}\right) \end{array}\right\},$ ∀z∈Z.

**Example** **10.** *Consider iv-FHSS $\left({{\tilde{F}}^{ivf}}_{HS}^{1},\Omega \right)$ as in Example 5. Partial membership of iv-FHSS $\left({{\tilde{F}}^{ivf}}_{HS}^{1},\Omega \right)$ is calculated as follows: $⊡\left({{\tilde{F}}^{ivf}}_{HS}^{1},\Omega_{1}\right)=\left\{\begin{array}{c} \left(\omega_{1},\frac{\left[0.4,0.6\right]}{z_{1}},\frac{\left[0.2,0.8\right]}{z_{2}},\frac{\left[0.5,0.5\right]}{z_{3}},\frac{\left[0.4,0.6\right]}{z_{4}},\frac{\left[0.3,0.7\right]}{z_{5}}\right),\left(\omega_{2},\frac{\left[0.3,0.7\right]}{z_{1}},\frac{\left[0.2,0.8\right]}{z_{2}},\frac{\left[0.2,0.8\right]}{z_{3}},\frac{\left[0.4,0.6\right]}{z_{4}},\frac{\left[0.2,0.8\right]}{z_{5}}\right)\\ \left(\omega_{3},\frac{\left[0.3,0.7\right]}{z_{1}},\frac{\left[0.1,0.9\right]}{z_{2}},\frac{\left[0.3,0.7\right]}{z_{3}},\frac{\left[0.2,0.8\right]}{z_{4}},\frac{\left[0.3,0.7\right]}{z_{5}}\right),\left(\omega_{4},\frac{\left[0.2,0.8\right]}{z_{1}},\frac{\left[0.4,0.6\right]}{z_{2}},\frac{\left[0.2,0.8\right]}{z_{3}},\frac{\left[0.3,0.7\right]}{z_{4}},\frac{\left[0.1,0.9\right]}{z_{5}}\right)\\ \left(\omega_{5},\frac{\left[0.3,0.7\right]}{z_{1}},\frac{\left[0.2,0.8\right]}{z_{2}},\frac{\left[0.1,0.9\right]}{z_{3}},\frac{\left[0.2,0.8\right]}{z_{4}},\frac{\left[0.1,0.9\right]}{z_{5}}\right),\left(\omega_{6},\frac{\left[0.4,0.6\right]}{z_{1}},\frac{\left[0.1,0.9\right]}{z_{2}},\frac{\left[0.4,0.6\right]}{z_{3}},\frac{\left[0.2,0.8\right]}{z_{4}},\frac{\left[0.3,0.7\right]}{z_{5}}\right)\\ \left(\omega_{7},\frac{\left[0.1,0.9\right]}{z_{1}},\frac{\left[0.1,0.9\right]}{z_{2}},\frac{\left[0.2,0.8\right]}{z_{3}},\frac{\left[0.4,0.6\right]}{z_{4}},\frac{\left[0.3,0.7\right]}{z_{5}}\right),\left(\omega_{8},\frac{\left[0.5,0.5\right]}{z_{1}},\frac{\left[0.2,0.8\right]}{z_{2}},\frac{\left[0.3,0.7\right]}{z_{3}},\frac{\left[0.2,0.8\right]}{z_{4}},\frac{\left[0.1,0.9\right]}{z_{5}}\right) \end{array}\right\},$ ∀z∈Z.*

**Example** **11.** *Consider iv-FHSS $\left({{\tilde{F}}^{ivf}}_{HS}^{1},\Omega \right)$ as in Example 5. Partial non-membership of iv-FHSS $\left({{\tilde{F}}^{ivf}}_{HS}^{1},\Omega \right)$ is as follows: $⊚\left({{\tilde{F}}^{ivf}}_{HS}^{1},\Omega_{1}\right)=\left\{\begin{array}{c} \left(\omega_{1},\frac{\left[0.3,0.7\right]}{z_{1}},\frac{\left[0.3,0.7\right]}{z_{2}},\frac{\left[0.4,0.6\right]}{z_{3}},\frac{\left[0.5,0.5\right]}{z_{4}},\frac{\left[0.4,0.6\right]}{z_{5}}\right),\left(\omega_{2},\frac{\left[0.5,0.5\right]}{z_{1}},\frac{\left[0.4,0.6\right]}{z_{2}},\frac{\left[0.2,0.8\right]}{z_{3}},\frac{\left[0.5,0.5\right]}{z_{4}},\frac{\left[0.4,0.6\right]}{z_{5}}\right)\\ \left(\omega_{3},\frac{\left[0.5,0.5\right]}{z_{1}},\frac{\left[0.4,0.6\right]}{z_{2}},\frac{\left[0.4,0.6\right]}{z_{3}},\frac{\left[0.4,0.6\right]}{z_{4}},\frac{\left[0.5,0.5\right]}{z_{5}}\right),\left(\omega_{4},\frac{\left[0.4,0.6\right]}{z_{1}},\frac{\left[0.4,0.6\right]}{z_{2}},\frac{\left[0.4,0.6\right]}{z_{3}},\frac{\left[0.3,0.7\right]}{z_{4}},\frac{\left[0.5,0.5\right]}{z_{5}}\right)\\ \left(\omega_{5},\frac{\left[0.5,0.5\right]}{z_{1}},\frac{\left[0.4,0.6\right]}{z_{2}},\frac{\left[0.4,0.6\right]}{z_{3}},\frac{\left[0.2,0.8\right]}{z_{4}},\frac{\left[0.4,0.6\right]}{z_{5}}\right),\left(\omega_{6},\frac{\left[0.5,0.5\right]}{z_{1}},\frac{\left[0.3,0.7\right]}{z_{2}},\frac{\left[0.3,0.7\right]}{z_{3}},\frac{\left[0.5,0.5\right]}{z_{4}},\frac{\left[0.3,0.7\right]}{z_{5}}\right)\\ \left(\omega_{7},\frac{\left[0.4,0.6\right]}{z_{1}},\frac{\left[0.5,0.5\right]}{z_{2}},\frac{\left[0.4,0.6\right]}{z_{3}},\frac{\left[0.5,0.5\right]}{z_{4}},\frac{\left[0.4,0.6\right]}{z_{5}}\right),\left(\omega_{8},\frac{\left[0.1,0.9\right]}{z_{1}},\frac{\left[0.5,0.5\right]}{z_{2}},\frac{\left[0.3,0.7\right]}{z_{3}},\frac{\left[0.5,0.5\right]}{z_{4}},\frac{\left[0.4,0.6\right]}{z_{5}}\right) \end{array}\right\},$ ∀z∈Z.*

## 4. Distance Measures between Interval-Valued Fuzzy Hypersoft Sets

In this section, the distance measures of iv-FHSSs are discussed.

Let $\left({\tilde{F}}_{HS}^{ivf},E\right)$ be an iv-FHSS over Z; then, for each η∈E approximate set ${\tilde{F}}_{HS}^{ivf}\left(\eta \right)$ is given by ${\tilde{F}}_{HS}^{ivf}\left(z\right)=\left\{(z,\kappa_{{\tilde{F}}_{HS}^{ivf}\left(\eta \right)}\left(z\right))\right\}$ where $\kappa_{{\tilde{F}}_{HS}^{ivf}\left(\eta \right)}\left(z\right)=\left[\kappa_{l{\tilde{F}}_{HS}^{ivf}\left(\eta \right)}\left(z\right),\kappa_{u{\tilde{F}}_{HS}^{ivf}\left(\eta \right)}\left(z\right)\right]$. Euclidean distance measure $d_{Euc}$, Hamming distance measure $d_{Ham}$ and Hausdorff distance measure $d_{Hau}$ for two iv-FHSSs are given as follows:

**Definition** **9.** *Let Euclidean distance between iv-FHSSs $\left({{\tilde{F}}^{ivf}}_{HS}^{1},\Lambda_{1}\right)$ and $\left({{\tilde{F}}^{ivf}}_{HS}^{2},\Lambda_{2}\right)$ be denoted by*

dEucF˜ivfHS1,Λ1,F˜ivfHS2,Λ2=∑η∈EdEuc*F˜ivfHS1(η),F˜ivfHS2(η)∑e∈Edann

where $d_{Euc}^{*}\left(\left({{\tilde{F}}^{ivf}}_{HS}^{1},\Lambda_{1}\right),\left({{\tilde{F}}^{ivf}}_{HS}^{2},\Lambda_{2}\right)\right)$ is given by $d_{Euc}^{*}\left(\left({{\tilde{F}}^{ivf}}_{HS}^{1},\Lambda_{1}\right),\left({{\tilde{F}}^{ivf}}_{HS}^{2},\Lambda_{2}\right)\right)=\left(1/2\right)\Sigma_{i=1}^{m}{\left({\left({𝒦}_{l{{\tilde{F}}^{ivf}}_{HS}^{1}\left(\eta \right)}\left(z_{i}\right)-{𝒦}_{l{{\tilde{F}}^{ivf}}_{HS}^{2}\left(\eta \right)}\left(z_{i}\right)\right)}^{2}+{\left({𝒦}_{u{{\tilde{F}}^{ivf}}_{HS}^{1}\left(\eta \right)}\left(z_{i}\right)-{𝒦}_{u{{\tilde{F}}^{ivf}}_{HS}^{2}\left(\eta \right)}\left(z_{i}\right)\right)}^{2}\right)}^{\frac{1}{2}}$ *such that $\eta \in \Lambda_{1}\cap \Lambda_{2}.$*

**Definition** **10.** *Let Hamming distance between iv-FHSSs $\left({{\tilde{F}}^{ivf}}_{HS}^{1},\Lambda_{1}\right)$ and $\left({{\tilde{F}}^{ivf}}_{HS}^{2},\Lambda_{2}\right)$ be denoted by*

dHamF˜ivfHS1,Λ1,F˜ivfHS2,Λ2=∑η∈EdHam*F˜ivfHS1(η),F˜ivfHS2(η)∑e∈Edann

where $d_{Ham}^{*}\left(\left({{\tilde{F}}^{ivf}}_{HS}^{1},\Lambda_{1}\right),\left({{\tilde{F}}^{ivf}}_{HS}^{2},\Lambda_{2}\right)\right)$ is given by $d_{Ham}^{*}\left(\left({{\tilde{F}}^{ivf}}_{HS}^{1},\Lambda_{1}\right),\left({{\tilde{F}}^{ivf}}_{HS}^{2},\Lambda_{2}\right)\right)=\left(1/2\right)\Sigma_{i=1}^{m}\left(\left|{𝒦}_{l{{\tilde{F}}^{ivf}}_{HS}^{1}\left(\eta \right)}\left(z_{i}\right)-{𝒦}_{l{{\tilde{F}}^{ivf}}_{HS}^{2}\left(\eta \right)}\left(z_{i}\right)\right|+\left|{𝒦}_{u{{\tilde{F}}^{ivf}}_{HS}^{1}\left(\eta \right)}\left(z_{i}\right)-{𝒦}_{u{{\tilde{F}}^{ivf}}_{HS}^{2}\left(\eta \right)}\left(z_{i}\right)\right|\right)$ *such that $\eta \in \Lambda_{1}\cap \Lambda_{2}.$*

**Definition** **11.** *Let Hausdorff distance between iv-FHSSs $\left({{\tilde{F}}^{ivf}}_{HS}^{1},\Lambda_{1}\right)$ and $\left({{\tilde{F}}^{ivf}}_{HS}^{2},\Lambda_{2}\right)$ be denoted by*

dHauF˜ivfHS1,Λ1,F˜ivfHS2,Λ2=∑η∈EdHau*F˜ivfHS1(η),F˜ivfHS2(η)∑e∈Edann

where $d_{Hau}^{*}\left(\left({{\tilde{F}}^{ivf}}_{HS}^{1},\Lambda_{1}\right),\left({{\tilde{F}}^{ivf}}_{HS}^{2},\Lambda_{2}\right)\right)$ is given by $d_{Hau}^{*}\left(\left({{\tilde{F}}^{ivf}}_{HS}^{1},\Lambda_{1}\right),\left({{\tilde{F}}^{ivf}}_{HS}^{2},\Lambda_{2}\right)\right)=$

$\max_{i}\left\{\left|\kappa_{l{{\tilde{F}}^{ivf}}_{HS}^{1}\left(\eta \right)}\left(z_{i}\right)-\kappa_{l{{\tilde{F}}^{ivf}}_{HS}^{2}\left(\eta \right)}\left(z_{i}\right)\right|,\left|\kappa_{u{{\tilde{F}}^{ivf}}_{HS}^{1}\left(\eta \right)}\left(z_{i}\right)-\kappa_{u{{\tilde{F}}^{ivf}}_{HS}^{2}\left(\eta \right)}\left(z_{i}\right)\right|\right\}$ *such that $\eta \in \Lambda_{1}\cap \Lambda_{2}.$*

## 5. Proposed Algorithm and Implementation

**Figure 1:**
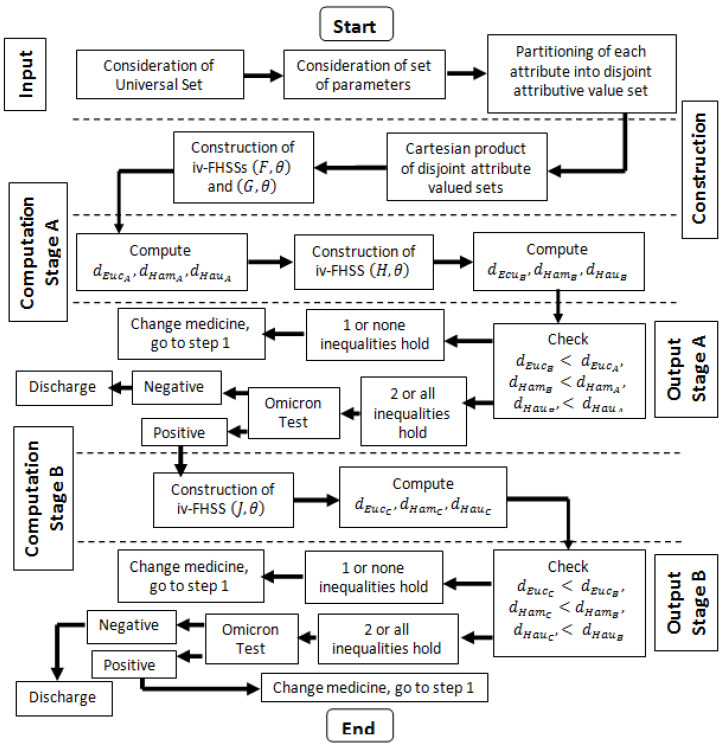
Flowchart: treatment of Omicron patient.

The following algorithm i.e., Algorithm 1 is developed by using iv-FHSS for decision making.
**Algorithm 1:** The assessment of medication effects in Omicron patients (see Figure 1 for flowchart)▹** *Start***▹** *Input Stage* **: —*1. Consider*
Z
*as universe of discourse.* —*2. Consider*
$\Theta^{1}$
*as subset of set of parameters.* —*3. Classify parameters into disjoint parametric valued sets*
$\Theta_{1},\Theta_{2},\Theta_{3}$.▹** *Construction Stage* **: —*4. Construct*
$\Theta =\Theta_{1}\times \Theta_{2}\times \Theta_{3}$. —*5. Construct iv-FHSSs*
$\left({{\tilde{F}}^{ivf}}_{HS},\Theta \right)$, $\left({{\tilde{G}}^{ivf}}_{HS},\Theta \right)$▹** *Computation Stage A* **: —*6. Compute*
$d_{Euc_{A}}=d_{Euc}\left(\left({{\tilde{F}}^{ivf}}_{HS},\Theta \right),\left({{\tilde{G}}^{ivf}}_{HS},\Theta \right)\right)$. —*7. Compute*
$d_{Ham_{A}}=d_{Ham}\left(\left({{\tilde{F}}^{ivf}}_{HS},\Theta \right),\left({{\tilde{G}}^{ivf}}_{HS},\Theta \right)\right)$. —*8. Compute*
$d_{Hau_{A}}=d_{Hau}\left(\left({{\tilde{F}}^{ivf}}_{HS},\Theta \right),\left({{\tilde{G}}^{ivf}}_{HS},\Theta \right)\right)$. —*9. Construct iv-FHSSs*$\left({{\tilde{H}}^{ivf}}_{HS},\Theta \right)$ —*10. Compute*
$d_{Euc_{B}},d_{Ham_{B}}$
*and*
$d_{Hau_{B}}$.▹** *Output Stage A* **: —*11. If*
$d_{Euc_{B}}<d_{Euc_{A}}$*,*
$d_{Ham_{B}}<d_{Ham_{A}}$
*and*
$d_{Hau_{B}}<d_{Hau_{A}}$
*hold or if at least two * *inequalities hold then the patient is recovering and needs an omicron test. If the test report * *is negative then stop medication and discharge; otherwise, go to next stage.* —*12. If one inequality holds or all inequalities become untrue then change medicine and * *go to stage I.*▹** *Computation Stage B* **: —*13. Construct iv-FHSSs*
$\left({{\tilde{J}}^{ivf}}_{HS},\Theta \right)$ —*14. Compute*
$d_{Euc_{C}},d_{Ham_{C}}$
*and*
$d_{Hau_{C}}$.▹** *Output Stage B* **: —*15. If*
$d_{Euc_{C}}<d_{Euc_{B}}$*,*
$d_{Ham_{C}}<d_{Ham_{B}}$
*and*
$d_{Hau_{C}}<d_{Hau_{B}}$
*hold or if at least two * *inequalities hold then the patient is recovering and needs omicron test. If test report is * *negative then stop medication and discharge otherwise repeat medication.* —*16. If one inequality hold or all inequalities become untrue then change medicine and*
*go to stage I.*▹** *End* **

### 5.1. Application

A brief introduction to the outbreak of the Omicron variant is discussed in this section along with the optimised effect of medication on the treatment of Omicron patients.

### 5.2. Outbreak of Omicron Variant

The first confirmed case of Omicron [36] was reported in the second week of November last year as per data collected by WHO. Since that time, the distinct proof of the Omicron variant appeared to quickly spread. A new genomic-arrangement investigation on 77 infection tests gathered in South Africa in mid-November indicated an alarming situation, as all the investigated variations were of Omicron [37]. The average number of cases grew from roughly 300 to 900 daily, and reached the milestone of 2000 in the last week of November 2021 [38]. Positive cases of this variant escalated so much that WHO upgraded its category from VM to VC in just two days. Within a short while, this variant spread to more than 35 nations across Europe, Africa and America. A lot of work is to be performed on research into how and where this variation initially developed. Among three major waves of COVID-19 from June 2020 to December 2021, *β* and *γ* variations are responsible for two waves in South Africa. Research [38] showed that the spread the of *β* variant was almost 50% of day-to-day diseases and increased up to 80% for the *δ* variant; however, in the case of Omicron, the level increased up to 90% in just the last month of 2021. These results show the dominance of Omicron over other variants.

### 5.3. Optimised Effect of Medication on Treatment of Omicron Patient

Suppose there are five patients diagnosed with Omicron who form the set of universe $Z=\left\{z_{1},z_{2},\ldots ,z_{5}\right\}$. Among all the symptoms of Omicron, some symptoms, such as fever, tiredness, cough and sensory loss, are most common, but there are other symptoms which are less common in patients, such as rashes on skin, sore throat, irritated eyes, headache, discolouration of fingers, diarrhoea, aches and pains. A team of health-care professionals was assigned the duty of collection and interpretation of data. The team considered only the most common symptoms, so the set of attributes consists of the most common symptoms, i.e., $\Theta_{1}=$ fever, $\Theta_{2}=$ cough, $\Theta_{3}=$ tiredness and $\Theta_{4}=$ sensory loss. Therefore, $E=\left\{\Theta_{1},\Theta_{2},\Theta_{3},\Theta_{4}\right\}$. A person with a body temperature between 99.5 F to 100.4 F is considered to have a low-grade fever. According to U.S. Centers for Disease Control and Prevention (CDC), a temperature at or above 100.4 F is considered as a high fever, so $\Theta_{1}=$ {low fever, high fever} $=\left\{\theta_{11},\theta_{12}\right\}$. According to a research by Hsu et al. [39], 0 to 16 coughs per day were recorded for a healthy individual, whereas above this range was considered as high coughing, so $\Theta_{2}=$ {low coughing, high coughing} $=\left\{\theta_{21},\theta_{22}\right\}$. $\Theta_{3}=$ {tiredness} $=\left\{\theta_{31}\right\}$. $\Theta_{4}=$ {loss of smell, loss of taste} $=\left\{\theta_{41},\theta_{42}\right\}$. To construct iv-FHSSs, the cartesian product of disjoint attributive sets is needed. Therefore, $\begin{array}{c} \Theta =\Theta_{1}\times \Theta_{2}\times \Theta_{3}\times \Theta_{4}\\ =\left\{\begin{array}{c} \left(\theta_{11},\theta_{21},\theta_{31},\theta_{41}\right),\left(\theta_{11},\theta_{21},\theta_{31},\theta_{42}\right),\left(\theta_{11},\theta_{22},\theta_{31},\theta_{41}\right),\left(\theta_{11},\theta_{22},\theta_{31},\theta_{42}\right),\\ \left(\theta_{12},\theta_{21},\theta_{31},\theta_{41}\right),\left(\theta_{12},\theta_{21},\theta_{31},\theta_{42}\right),\left(\theta_{12},\theta_{22},\theta_{31},\theta_{41}\right),\left(\theta_{12},\theta_{22},\theta_{31},\theta_{42}\right) \end{array}\right\}.\\ =\left\{\theta_{1},\theta_{2},\theta_{3},\theta_{4},\theta_{5},\theta_{6},\theta_{7},\theta_{8}\right\} \end{array}$

The treatment process of the patients is divided into four stages to evaluate the stepwise improvement in the patients.

Stage I: Premedication.
Stage II: Phase 1 medication.
Stage III: Phase 2 medication.
Stage IV: Post-medication.

In Stage I, data is collected from patients which shows how they feel and they are given treatment against the virus for five days. The Omicron test is taken and the same form is completed by patients whose tests result is positive. In Stage III, patients are treated again for five days and an Omicron test is taken. The patients having a positive report of the virus are treated for five days after completing the form. At the end of 15 days, a final Omicron test is taken and form completed. The patients having a negative test report at any stage are discharged. All those patients who still have symptoms of the virus are re-medicated from Stage I. The complete process of treatment is described a follows: A form was completed by each patient before medication during their treatment which shows how they feel. According to the information provided by them, iv-FHSS ${{\tilde{F}}^{ivf}}_{HS}\left(\Theta \right)$ is constructed and given as ${{\tilde{F}}^{ivf}}_{HS}\left(\Theta \right)=\left\{{{\tilde{F}}^{ivf}}_{HS}\left(\theta_{1}\right),{{\tilde{F}}^{ivf}}_{HS}\left(\theta_{2}\right),\ldots ,{{\tilde{F}}^{ivf}}_{HS}\left(\theta_{8}\right)\right\}$ with

$${{\tilde{F}}^{ivf}}_{HS}\left(\theta_{1}\right)=\left\{\frac{\left[0.6,0.7\right]}{z_{1}},\frac{\left[0.2,0.3\right]}{z_{2}},\frac{\left[0.5,0.6\right]}{z_{3}},\frac{\left[0.4,0.5\right]}{z_{4}},\frac{\left[0.3,0.4\right]}{z_{5}}\right\},$$

$${{\tilde{F}}^{ivf}}_{HS}\left(\theta_{2}\right)=\left\{\frac{\left[0.3,0.5\right]}{z_{1}},\frac{\left[0.2,0.4\right]}{z_{2}},\frac{\left[0.2,0.8\right]}{z_{3}},\frac{\left[0.4,0.5\right]}{z_{4}},\frac{\left[0.2,0.6\right]}{z_{5}}\right\},$$

$${{\tilde{F}}^{ivf}}_{HS}\left(\theta_{3}\right)=\left\{\frac{\left[0.3,0.5\right]}{z_{1}},\frac{\left[0.1,0.4\right]}{z_{2}},\frac{\left[0.3,0.6\right]}{z_{3}},\frac{\left[0.2,0.6\right]}{z_{4}},\frac{\left[0.3,0.5\right]}{z_{5}}\right\},$$

$${{\tilde{F}}^{ivf}}_{HS}\left(\theta_{4}\right)=\left\{\frac{\left[0.2,0.6\right]}{z_{1}},\frac{\left[0.4,0.6\right]}{z_{2}},\frac{\left[0.2,0.4\right]}{z_{3}},\frac{\left[0.3,0.7\right]}{z_{4}},\frac{\left[0.1,0.5\right]}{z_{5}}\right\},$$

$${{\tilde{F}}^{ivf}}_{HS}\left(\theta_{5}\right)=\left\{\frac{\left[0.3,0.5\right]}{z_{1}},\frac{\left[0.2,0.6\right]}{z_{2}},\frac{\left[0.1,0.4\right]}{z_{3}},\frac{\left[0.2,0.8\right]}{z_{4}},\frac{\left[0.1,0.4\right]}{z_{5}}\right\},$$

$${{\tilde{F}}^{ivf}}_{HS}\left(\theta_{6}\right)=\left\{\frac{\left[0.4,0.5\right]}{z_{1}},\frac{\left[0.1,0.3\right]}{z_{2}},\frac{\left[0.4,0.7\right]}{z_{3}},\frac{\left[0.2,0.5\right]}{z_{4}},\frac{\left[0.3,0.7\right]}{z_{5}}\right\},$$

$${{\tilde{F}}^{ivf}}_{HS}\left(\theta_{7}\right)=\left\{\frac{\left[0.1,0.4\right]}{z_{1}},\frac{\left[0.1,0.5\right]}{z_{2}},\frac{\left[0.2,0.6\right]}{z_{3}},\frac{\left[0.4,0.5\right]}{z_{4}},\frac{\left[0.3,0.4\right]}{z_{5}}\right\},$$

$${{\tilde{F}}^{ivf}}_{HS}\left(\theta_{8}\right)=\left\{\frac{\left[0.5,0.9\right]}{z_{1}},\frac{\left[0.2,0.5\right]}{z_{2}},\frac{\left[0.3,0.7\right]}{z_{3}},\frac{\left[0.2,0.5\right]}{z_{4}},\frac{\left[0.1,0.4\right]}{z_{5}}\right\}.$$

The iv-FHSS $\left({{\tilde{F}}^{ivf}}_{HS},\Theta \right)$ can also be written as $\left({{\tilde{F}}^{ivf}}_{HS},\Theta \right)$ = $\left\{\begin{array}{c} \left(\theta_{1},\frac{\left[0.6,0.7\right]}{z_{1}},\frac{\left[0.2,0.3\right]}{z_{2}},\frac{\left[0.5,0.6\right]}{z_{3}},\frac{\left[0.4,0.5\right]}{z_{4}},\frac{\left[0.3,0.4\right]}{z_{5}}\right),\left(\theta_{2},\frac{\left[0.3,0.5\right]}{z_{1}},\frac{\left[0.2,0.4\right]}{z_{2}},\frac{\left[0.2,0.8\right]}{z_{3}},\frac{\left[0.4,0.5\right]}{z_{4}},\frac{\left[0.2,0.6\right]}{z_{5}}\right)\\ \left(\theta_{3},\frac{\left[0.3,0.5\right]}{z_{1}},\frac{\left[0.1,0.4\right]}{z_{2}},\frac{\left[0.3,0.6\right]}{z_{3}},\frac{\left[0.2,0.6\right]}{z_{4}},\frac{\left[0.3,0.5\right]}{z_{5}}\right),\left(\theta_{4},\frac{\left[0.2,0.6\right]}{z_{1}},\frac{\left[0.4,0.6\right]}{z_{2}},\frac{\left[0.2,0.4\right]}{z_{3}},\frac{\left[0.3,0.7\right]}{z_{4}},\frac{\left[0.1,0.5\right]}{z_{5}}\right)\\ \left(\theta_{5},\frac{\left[0.3,0.5\right]}{z_{1}},\frac{\left[0.2,0.6\right]}{z_{2}},\frac{\left[0.1,0.4\right]}{z_{3}},\frac{\left[0.2,0.8\right]}{z_{4}},\frac{\left[0.1,0.4\right]}{z_{5}}\right),\left(\theta_{6},\frac{\left[0.4,0.5\right]}{z_{1}},\frac{\left[0.1,0.3\right]}{z_{2}},\frac{\left[0.4,0.7\right]}{z_{3}},\frac{\left[0.2,0.5\right]}{z_{4}},\frac{\left[0.3,0.7\right]}{z_{5}}\right)\\ \left(\theta_{7},\frac{\left[0.1,0.4\right]}{z_{1}},\frac{\left[0.1,0.5\right]}{z_{2}},\frac{\left[0.2,0.6\right]}{z_{3}},\frac{\left[0.4,0.5\right]}{z_{4}},\frac{\left[0.3,0.4\right]}{z_{5}}\right),\left(\theta_{8},\frac{\left[0.5,0.9\right]}{z_{1}},\frac{\left[0.2,0.5\right]}{z_{2}},\frac{\left[0.3,0.7\right]}{z_{3}},\frac{\left[0.2,0.5\right]}{z_{4}},\frac{\left[0.1,0.4\right]}{z_{5}}\right) \end{array}\right\}$

After treatment for 5 days, the same form was completed by each patient and information in the form used for iv-FHSS ${{\tilde{G}}^{ivf}}_{HS}\left(\Theta \right)$, which is given as ${{\tilde{G}}^{ivf}}_{HS}\left(\Theta \right)=\{{{\tilde{G}}^{ivf}}_{HS}\left(\theta_{1}\right),{{\tilde{G}}^{ivf}}_{HS}\left(\theta_{2}\right),\ldots ,$${{\tilde{G}}^{ivf}}_{HS}\left(\theta_{8}\right)\}$ with

$${{\tilde{G}}^{ivf}}_{HS}\left(\theta_{1}\right)=\left\{\frac{\left[0.3,0.4\right]}{z_{1}},\frac{\left[0.3,0.6\right]}{z_{2}},\frac{\left[0.3,0.5\right]}{z_{3}},\frac{\left[0.2,0.5\right]}{z_{4}},\frac{\left[0.3,0.6\right]}{z_{5}}\right\},$$

$${{\tilde{G}}^{ivf}}_{HS}\left(\theta_{2}\right)=\left\{\frac{\left[0.4,0.6\right]}{z_{1}},\frac{\left[0.2,0.5\right]}{z_{2}},\frac{\left[0.4,0.7\right]}{z_{3}},\frac{\left[0.3,0.5\right]}{z_{4}},\frac{\left[0.5,0.7\right]}{z_{5}}\right\},$$

$${{\tilde{G}}^{ivf}}_{HS}\left(\theta_{3}\right)=\left\{\frac{\left[0.4,0.6\right]}{z_{1}},\frac{\left[0.1,0.5\right]}{z_{2}},\frac{\left[0.5,0.7\right]}{z_{3}},\frac{\left[0.4,0.6\right]}{z_{4}},\frac{\left[0.3,0.6\right]}{z_{5}}\right\},$$

$${{\tilde{G}}^{ivf}}_{HS}\left(\theta_{4}\right)=\left\{\frac{\left[0.2,0.6\right]}{z_{1}},\frac{\left[0.4,0.7\right]}{z_{2}},\frac{\left[0.1,0.3\right]}{z_{3}},\frac{\left[0.4,0.8\right]}{z_{4}},\frac{\left[0.2,0.5\right]}{z_{5}}\right\},$$

$${{\tilde{G}}^{ivf}}_{HS}\left(\theta_{5}\right)=\left\{\frac{\left[0.4,0.5\right]}{z_{1}},\frac{\left[0.3,0.6\right]}{z_{2}},\frac{\left[0.3,0.4\right]}{z_{3}},\frac{\left[0.4,0.6\right]}{z_{4}},\frac{\left[0.3,0.5\right]}{z_{5}}\right\},$$

$${{\tilde{G}}^{ivf}}_{HS}\left(\theta_{6}\right)=\left\{\frac{\left[0.4,0.5\right]}{z_{1}},\frac{\left[0.3,0.5\right]}{z_{2}},\frac{\left[0.5,0.6\right]}{z_{3}},\frac{\left[0.3,0.6\right]}{z_{4}},\frac{\left[0.4,0.8\right]}{z_{5}}\right\},$$

$${{\tilde{G}}^{ivf}}_{HS}\left(\theta_{7}\right)=\left\{\frac{\left[0.3,0.5\right]}{z_{1}},\frac{\left[0.2,0.6\right]}{z_{2}},\frac{\left[0.3,0.6\right]}{z_{3}},\frac{\left[0.2,0.4\right]}{z_{4}},\frac{\left[0.5,0.7\right]}{z_{5}}\right\},$$

$${{\tilde{G}}^{ivf}}_{HS}\left(\theta_{8}\right)=\left\{\frac{\left[0.7,0.9\right]}{z_{1}},\frac{\left[0.4,0.6\right]}{z_{2}},\frac{\left[0.4,0.8\right]}{z_{3}},\frac{\left[0.3,0.5\right]}{z_{4}},\frac{\left[0.3,0.6\right]}{z_{5}}\right\}.$$

The iv-FHSS $\left({{\tilde{G}}^{ivf}}_{HS},\Theta \right)$ can also be written as $\left({{\tilde{G}}^{ivf}}_{HS},\Theta \right)$ = $\left\{\begin{array}{c} \left(\theta_{1},\frac{\left[0.3,0.4\right]}{z_{1}},\frac{\left[0.3,0.6\right]}{z_{2}},\frac{\left[0.3,0.5\right]}{z_{3}},\frac{\left[0.2,0.5\right]}{z_{4}},\frac{\left[0.3,0.6\right]}{z_{5}}\right),\left(\theta_{2},\frac{\left[0.4,0.6\right]}{z_{1}},\frac{\left[0.2,0.5\right]}{z_{2}},\frac{\left[0.4,0.7\right]}{z_{3}},\frac{\left[0.3,0.5\right]}{z_{4}},\frac{\left[0.5,0.7\right]}{z_{5}}\right)\\ \left(\theta_{3},\frac{\left[0.4,0.6\right]}{z_{1}},\frac{\left[0.1,0.5\right]}{z_{2}},\frac{\left[0.5,0.7\right]}{z_{3}},\frac{\left[0.4,0.6\right]}{z_{4}},\frac{\left[0.3,0.6\right]}{z_{5}}\right),\left(\theta_{4},\frac{\left[0.2,0.6\right]}{z_{1}},\frac{\left[0.4,0.7\right]}{z_{2}},\frac{\left[0.1,0.3\right]}{z_{3}},\frac{\left[0.4,0.8\right]}{z_{4}},\frac{\left[0.2,0.5\right]}{z_{5}}\right)\\ \left(\theta_{5},\frac{\left[0.4,0.5\right]}{z_{1}},\frac{\left[0.3,0.6\right]}{z_{2}},\frac{\left[0.3,0.4\right]}{z_{3}},\frac{\left[0.4,0.6\right]}{z_{4}},\frac{\left[0.3,0.5\right]}{z_{5}}\right),\left(\theta_{6},\frac{\left[0.4,0.5\right]}{z_{1}},\frac{\left[0.3,0.5\right]}{z_{2}},\frac{\left[0.5,0.6\right]}{z_{3}},\frac{\left[0.3,0.6\right]}{z_{4}},\frac{\left[0.4,0.8\right]}{z_{5}}\right)\\ \left(\theta_{7},\frac{\left[0.3,0.5\right]}{z_{1}},\frac{\left[0.2,0.6\right]}{z_{2}},\frac{\left[0.3,0.6\right]}{z_{3}},\frac{\left[0.2,0.4\right]}{z_{4}},\frac{\left[0.5,0.7\right]}{z_{5}}\right),\left(\theta_{8},\frac{\left[0.7,0.9\right]}{z_{1}},\frac{\left[0.4,0.6\right]}{z_{2}},\frac{\left[0.4,0.8\right]}{z_{3}},\frac{\left[0.3,0.5\right]}{z_{4}},\frac{\left[0.3,0.6\right]}{z_{5}}\right) \end{array}\right\}$

Euclidean, Hamming and Hausdorff distances are measured.

Euclidean distance between iv-FHSS $\left({{\tilde{F}}^{ivf}}_{HS},\Theta \right)$ and iv-FHSS $\left({{\tilde{G}}^{ivf}}_{HS},\Theta \right)$ can be calculated as:

$$d_{Euc}^{*}\left(\left({{\tilde{F}}^{ivf}}_{HS},\theta_{1}\right),\left({{\tilde{G}}^{ivf}}_{HS},\theta_{1}\right)\right)=0.6820,$$

$$d_{Euc}^{*}\left(\left({{\tilde{F}}^{ivf}}_{HS},\theta_{2}\right),\left({{\tilde{G}}^{ivf}}_{HS},\theta_{2}\right)\right)=0.4406,$$

$$d_{Euc}^{*}\left(\left({{\tilde{F}}^{ivf}}_{HS},\theta_{3}\right),\left({{\tilde{G}}^{ivf}}_{HS},\theta_{3}\right)\right)=0.3825,$$

$$d_{Euc}^{*}\left(\left({{\tilde{F}}^{ivf}}_{HS},\theta_{4}\right),\left({{\tilde{G}}^{ivf}}_{HS},\theta_{4}\right)\right)=0.2414,$$

$$d_{Euc}^{*}\left(\left({{\tilde{F}}^{ivf}}_{HS},\theta_{5}\right),\left({{\tilde{G}}^{ivf}}_{HS},\theta_{5}\right)\right)=0.4532,$$

$$d_{Euc}^{*}\left(\left({{\tilde{F}}^{ivf}}_{HS},\theta_{6}\right),\left({{\tilde{G}}^{ivf}}_{HS},\theta_{6}\right)\right)=0.3506,$$

$$d_{Euc}^{*}\left(\left({{\tilde{F}}^{ivf}}_{HS},\theta_{7}\right),\left({{\tilde{G}}^{ivf}}_{HS},\theta_{7}\right)\right)=0.5246,$$

$$d_{Euc}^{*}\left(\left({{\tilde{F}}^{ivf}}_{HS},\theta_{8}\right),\left({{\tilde{G}}^{ivf}}_{HS},\theta_{8}\right)\right)=0.4739,$$

so

$$d_{Euc_{A}}=d_{Euc}\left(\left({{\tilde{F}}^{ivf}}_{HS},\Theta \right),\left({{\tilde{G}}^{ivf}}_{HS},\Theta \right)\right)=0.4436$$

Hamming distance and Hausdorff distance can be calculated in a similar way, as follows:

$$d_{Ham_{A}}=d_{Ham}\left(\left({{\tilde{F}}^{ivf}}_{HS},\Theta \right),\left({{\tilde{G}}^{ivf}}_{HS},\Theta \right)\right)=0.5625,$$

$$d_{Hau_{A}}=d_{Hau}\left(\left({{\tilde{F}}^{ivf}}_{HS},\Theta \right),\left({{\tilde{G}}^{ivf}}_{HS},\Theta \right)\right)=0.2250$$

The Omicron test is taken. Patients with a negative test report are discharged. Patients with a positive test report are medicated for five more days. After treatment of five more days, the same form was completed by each patient and information in the form used for iv-FHSS ${{\tilde{H}}^{ivf}}_{HS}\left(\Theta \right)$, which is given as ${{\tilde{H}}^{ivf}}_{HS}\left(\Theta \right)=\left\{{{\tilde{H}}^{ivf}}_{HS}\left(\theta_{1}\right),{{\tilde{H}}^{ivf}}_{HS}\left(\theta_{2}\right),\ldots ,{{\tilde{H}}^{ivf}}_{HS}\left(\theta_{8}\right)\right\}$ with

$${{\tilde{H}}^{ivf}}_{HS}\left(\theta_{1}\right)=\left\{\frac{\left[0.3,0.5\right]}{z_{1}},\frac{\left[0.4,0.6\right]}{z_{2}},\frac{\left[0.3,0.5\right]}{z_{3}},\frac{\left[0.4,0.6\right]}{z_{4}},\frac{\left[0.4,0.5\right]}{z_{5}}\right\},$$

$${{\tilde{H}}^{ivf}}_{HS}\left(\theta_{2}\right)=\left\{\frac{\left[0.3,0.5\right]}{z_{1}},\frac{\left[0.4,0.6\right]}{z_{2}},\frac{\left[0.3,0.6\right]}{z_{3}},\frac{\left[0.4,0.5\right]}{z_{4}},\frac{\left[0.4,0.7\right]}{z_{5}}\right\},$$

$${{\tilde{H}}^{ivf}}_{HS}\left(\theta_{3}\right)=\left\{\frac{\left[0.3,0.5\right]}{z_{1}},\frac{\left[0.3,0.6\right]}{z_{2}},\frac{\left[0.6,0.8\right]}{z_{3}},\frac{\left[0.3,0.6\right]}{z_{4}},\frac{\left[0.5,0.7\right]}{z_{5}}\right\},$$

$${{\tilde{H}}^{ivf}}_{HS}\left(\theta_{4}\right)=\left\{\frac{\left[0.4,0.7\right]}{z_{1}},\frac{\left[0.3,0.8\right]}{z_{2}},\frac{\left[0.3,0.5\right]}{z_{3}},\frac{\left[0.5,0.7\right]}{z_{4}},\frac{\left[0.3,0.6\right]}{z_{5}}\right\},$$

$${{\tilde{H}}^{ivf}}_{HS}\left(\theta_{5}\right)=\left\{\frac{\left[0.3,0.6\right]}{z_{1}},\frac{\left[0.2,0.6\right]}{z_{2}},\frac{\left[0.2,0.4\right]}{z_{3}},\frac{\left[0.3,0.6\right]}{z_{4}},\frac{\left[0.1,0.4\right]}{z_{5}}\right\},$$

$${{\tilde{H}}^{ivf}}_{HS}\left(\theta_{6}\right)=\left\{\frac{\left[0.3,0.5\right]}{z_{1}},\frac{\left[0.4,0.5\right]}{z_{2}},\frac{\left[0.3,0.4\right]}{z_{3}},\frac{\left[0.3,0.5\right]}{z_{4}},\frac{\left[0.5,0.8\right]}{z_{5}}\right\},$$

$${{\tilde{H}}^{ivf}}_{HS}\left(\theta_{7}\right)=\left\{\frac{\left[0.4,0.6\right]}{z_{1}},\frac{\left[0.3,0.5\right]}{z_{2}},\frac{\left[0.5,0.7\right]}{z_{3}},\frac{\left[0.3,0.5\right]}{z_{4}},\frac{\left[0.4,0.7\right]}{z_{5}}\right\},$$

$${{\tilde{H}}^{ivf}}_{HS}\left(\theta_{8}\right)=\left\{\frac{\left[0.5,0.7\right]}{z_{1}},\frac{\left[0.3,0.5\right]}{z_{2}},\frac{\left[0.3,0.5\right]}{z_{3}},\frac{\left[0.4,0.6\right]}{z_{4}},\frac{\left[0.4,0.8\right]}{z_{5}}\right\}.$$

The iv-FHSS $\left({{\tilde{H}}^{ivf}}_{HS},\Theta \right)$ can also be written as $\left({{\tilde{H}}^{ivf}}_{HS},\Theta \right)$=

$\left\{\begin{array}{c} \left(\theta_{1},\frac{\left[0.3,0.5\right]}{z_{1}},\frac{\left[0.4,0.6\right]}{z_{2}},\frac{\left[0.3,0.5\right]}{z_{3}},\frac{\left[0.4,0.6\right]}{z_{4}},\frac{\left[0.4,0.5\right]}{z_{5}}\right),\left(\theta_{2},\frac{\left[0.3,0.5\right]}{z_{1}},\frac{\left[0.4,0.6\right]}{z_{2}},\frac{\left[0.3,0.6\right]}{z_{3}},\frac{\left[0.4,0.5\right]}{z_{4}},\frac{\left[0.4,0.7\right]}{z_{5}}\right)\\ \left(\theta_{3},\frac{\left[0.3,0.5\right]}{z_{1}},\frac{\left[0.3,0.6\right]}{z_{2}},\frac{\left[0.6,0.8\right]}{z_{3}},\frac{\left[0.3,0.6\right]}{z_{4}},\frac{\left[0.5,0.7\right]}{z_{5}}\right),\left(\theta_{4},\frac{\left[0.4,0.7\right]}{z_{1}},\frac{\left[0.3,0.8\right]}{z_{2}},\frac{\left[0.3,0.5\right]}{z_{3}},\frac{\left[0.5,0.7\right]}{z_{4}},\frac{\left[0.3,0.6\right]}{z_{5}}\right)\\ \left(\theta_{5},\frac{\left[0.3,0.6\right]}{z_{1}},\frac{\left[0.2,0.6\right]}{z_{2}},\frac{\left[0.2,0.4\right]}{z_{3}},\frac{\left[0.3,0.6\right]}{z_{4}},\frac{\left[0.1,0.4\right]}{z_{5}}\right),\left(\theta_{6},\frac{\left[0.3,0.5\right]}{z_{1}},\frac{\left[0.4,0.5\right]}{z_{2}},\frac{\left[0.3,0.4\right]}{z_{3}},\frac{\left[0.3,0.5\right]}{z_{4}},\frac{\left[0.5,0.8\right]}{z_{5}}\right)\\ \left(\theta_{7},\frac{\left[0.4,0.6\right]}{z_{1}},\frac{\left[0.3,0.5\right]}{z_{2}},\frac{\left[0.5,0.7\right]}{z_{3}},\frac{\left[0.3,0.5\right]}{z_{4}},\frac{\left[0.4,0.7\right]}{z_{5}}\right),\left(\theta_{8},\frac{\left[0.5,0.7\right]}{z_{1}},\frac{\left[0.3,0.5\right]}{z_{2}},\frac{\left[0.3,0.5\right]}{z_{3}},\frac{\left[0.4,0.6\right]}{z_{4}},\frac{\left[0.4,0.8\right]}{z_{5}}\right) \end{array}\right\}$

Euclidean, Hamming and Hausdorff distances are measured.

$$d_{Euc_{B}}=d_{Euc}\left(\left({{\tilde{G}}^{ivf}}_{HS},\Theta \right),\left({{\tilde{H}}^{ivf}}_{HS},\Theta \right)\right)=0.3896,$$

$$d_{Ham_{B}}=d_{Ham}\left(\left({{\tilde{G}}^{ivf}}_{HS},\Theta \right),\left({{\tilde{H}}^{ivf}}_{HS},\Theta \right)\right)=0.50625,$$

$$d_{Hau_{B}}=d_{Hau}\left(\left({{\tilde{G}}^{ivf}}_{HS},\Theta \right),\left({{\tilde{H}}^{ivf}}_{HS},\Theta \right)\right)=0.2125,$$

The decrease in distance measure shows improvement of patient’s health. An Omicron test is taken. Patients having a negative test report are discharged. Patients having a positive test report are medicated for five more days. After treatment of five more days, the same form was completed by each patient and the information in the form of iv-FHSS ${{\tilde{J}}^{ivf}}_{HS}\left(\Theta \right)$ is constructed, which is given as ${{\tilde{J}}^{ivf}}_{HS}\left(\Theta \right)=\left\{{{\tilde{J}}^{ivf}}_{HS}\left(\theta_{1}\right),{{\tilde{J}}^{ivf}}_{HS}\left(\theta_{2}\right),\ldots ,{{\tilde{J}}^{ivf}}_{HS}\left(\theta_{8}\right)\right\}$ with

$${{\tilde{J}}^{ivf}}_{HS}\left(\theta_{1}\right)=\left\{\frac{\left[0.4,0.5\right]}{z_{1}},\frac{\left[0.5,0.6\right]}{z_{2}},\frac{\left[0.5,0.6\right]}{z_{3}},\frac{\left[0.4,0.5\right]}{z_{4}},\frac{\left[0.4,0.5\right]}{z_{5}}\right\},$$

$${{\tilde{J}}^{ivf}}_{HS}\left(\theta_{2}\right)=\left\{\frac{\left[0.4,0.5\right]}{z_{1}},\frac{\left[0.5,0.6\right]}{z_{2}},\frac{\left[0.5,0.6\right]}{z_{3}},\frac{\left[0.4,0.5\right]}{z_{4}},\frac{\left[0.5,0.7\right]}{z_{5}}\right\},$$

$${{\tilde{J}}^{ivf}}_{HS}\left(\theta_{3}\right)=\left\{\frac{\left[0.4,0.6\right]}{z_{1}},\frac{\left[0.4,0.6\right]}{z_{2}},\frac{\left[0.6,0.8\right]}{z_{3}},\frac{\left[0.3,0.6\right]}{z_{4}},\frac{\left[0.5,0.7\right]}{z_{5}}\right\},$$

$${{\tilde{J}}^{ivf}}_{HS}\left(\theta_{4}\right)=\left\{\frac{\left[0.6,0.7\right]}{z_{1}},\frac{\left[0.6,0.8\right]}{z_{2}},\frac{\left[0.4,0.5\right]}{z_{3}},\frac{\left[0.6,0.7\right]}{z_{4}},\frac{\left[0.5,0.6\right]}{z_{5}}\right\},$$

$${{\tilde{J}}^{ivf}}_{HS}\left(\theta_{5}\right)=\left\{\frac{\left[0.5,0.6\right]}{z_{1}},\frac{\left[0.4,0.6\right]}{z_{2}},\frac{\left[0.3,0.5\right]}{z_{3}},\frac{\left[0.4,0.6\right]}{z_{4}},\frac{\left[0.3,0.4\right]}{z_{5}}\right\},$$

$${{\tilde{J}}^{ivf}}_{HS}\left(\theta_{6}\right)=\left\{\frac{\left[0.4,0.5\right]}{z_{1}},\frac{\left[0.4,0.5\right]}{z_{2}},\frac{\left[0.3,0.4\right]}{z_{3}},\frac{\left[0.4,0.5\right]}{z_{4}},\frac{\left[0.6,0.8\right]}{z_{5}}\right\},$$

$${{\tilde{J}}^{ivf}}_{HS}\left(\theta_{7}\right)=\left\{\frac{\left[0.5,0.6\right]}{z_{1}},\frac{\left[0.4,0.5\right]}{z_{2}},\frac{\left[0.5,0.6\right]}{z_{3}},\frac{\left[0.4,0.5\right]}{z_{4}},\frac{\left[0.5,0.7\right]}{z_{5}}\right\},$$

$${{\tilde{J}}^{ivf}}_{HS}\left(\theta_{8}\right)=\left\{\frac{\left[0.6,0.7\right]}{z_{1}},\frac{\left[0.4,0.5\right]}{z_{2}},\frac{\left[0.4,0.5\right]}{z_{3}},\frac{\left[0.5,0.6\right]}{z_{4}},\frac{\left[0.6,0.7\right]}{z_{5}}\right\}.$$

The iv-FHSS $\left({{\tilde{J}}^{ivf}}_{HS},\Theta \right)$ can also be written as $\left({{\tilde{J}}^{ivf}}_{HS},\Theta \right)$ = *$\left\{\begin{array}{c} \left(\theta_{1},\frac{\left[0.4,0.5\right]}{z_{1}},\frac{\left[0.5,0.6\right]}{z_{2}},\frac{\left[0.5,0.6\right]}{z_{3}},\frac{\left[0.4,0.5\right]}{z_{4}},\frac{\left[0.4,0.5\right]}{z_{5}}\right),\left(\theta_{2},\frac{\left[0.4,0.5\right]}{z_{1}},\frac{\left[0.5,0.6\right]}{z_{2}},\frac{\left[0.5,0.6\right]}{z_{3}},\frac{\left[0.4,0.5\right]}{z_{4}},\frac{\left[0.5,0.7\right]}{z_{5}}\right)\\ \left(\theta_{3},\frac{\left[0.4,0.6\right]}{z_{1}},\frac{\left[0.4,0.6\right]}{z_{2}},\frac{\left[0.6,0.8\right]}{z_{3}},\frac{\left[0.3,0.6\right]}{z_{4}},\frac{\left[0.5,0.7\right]}{z_{5}}\right),\left(\theta_{4},\frac{\left[0.6,0.7\right]}{z_{1}},\frac{\left[0.6,0.8\right]}{z_{2}},\frac{\left[0.4,0.5\right]}{z_{3}},\frac{\left[0.6,0.7\right]}{z_{4}},\frac{\left[0.5,0.6\right]}{z_{5}}\right)\\ \left(\theta_{5},\frac{\left[0.5,0.6\right]}{z_{1}},\frac{\left[0.4,0.6\right]}{z_{2}},\frac{\left[0.3,0.5\right]}{z_{3}},\frac{\left[0.4,0.6\right]}{z_{4}},\frac{\left[0.3,0.4\right]}{z_{5}}\right),\left(\theta_{6},\frac{\left[0.4,0.5\right]}{z_{1}},\frac{\left[0.4,0.5\right]}{z_{2}},\frac{\left[0.3,0.4\right]}{z_{3}},\frac{\left[0.4,0.5\right]}{z_{4}},\frac{\left[0.6,0.8\right]}{z_{5}}\right)\\ \left(\theta_{7},\frac{\left[0.5,0.6\right]}{z_{1}},\frac{\left[0.4,0.5\right]}{z_{2}},\frac{\left[0.5,0.6\right]}{z_{3}},\frac{\left[0.4,0.5\right]}{z_{4}},\frac{\left[0.5,0.7\right]}{z_{5}}\right),\left(\theta_{8},\frac{\left[0.6,0.7\right]}{z_{1}},\frac{\left[0.4,0.5\right]}{z_{2}},\frac{\left[0.4,0.5\right]}{z_{3}},\frac{\left[0.5,0.6\right]}{z_{4}},\frac{\left[0.6,0.7\right]}{z_{5}}\right) \end{array}\right\}$*

Euclidean, Hamming and Hausdorff distances are measured.

$$d_{Euc_{C}}=d_{Euc}\left(\left({{\tilde{H}}^{ivf}}_{HS},\Theta \right),\left({{\tilde{J}}^{ivf}}_{HS},\Theta \right)\right)=0.27765,$$

$$d_{Ham_{C}}=d_{Ham}\left(\left({{\tilde{H}}^{ivf}}_{HS},\Theta \right),\left({{\tilde{J}}^{ivf}}_{HS},\Theta \right)\right)=0.29375,$$

$$d_{Hau_{C}}=d_{Hau}\left(\left({{\tilde{H}}^{ivf}}_{HS},\Theta \right),\left({{\tilde{J}}^{ivf}}_{HS},\Theta \right)\right)=0.1750$$

The decrease in distance measure shows the improvement in a patient’s health. An Omicron test is taken. Patients having a negative test report are discharged. For patients having a positive test report, the process of medication is started from phase 1.

### 5.4. Discussion

**Figure 2:**
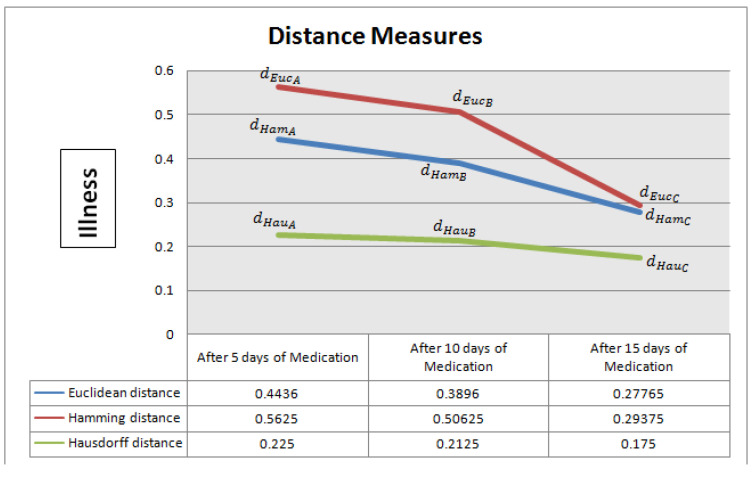
Graph: treatment of Omicron patients.

**Figure 3:**
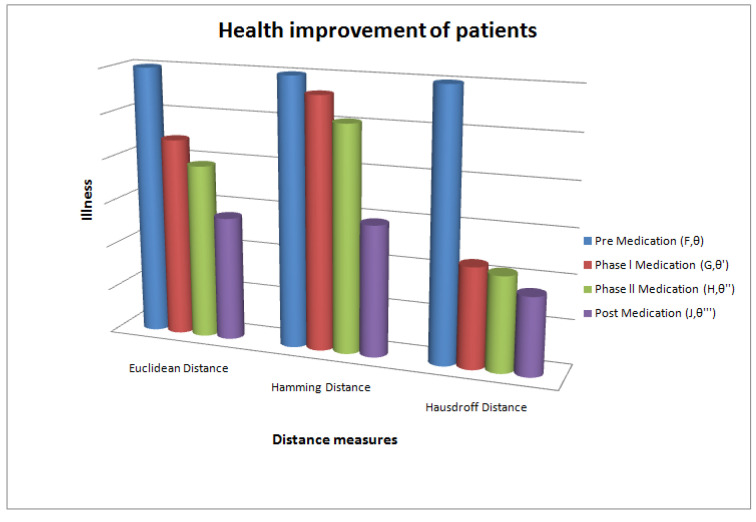
Graph: treatment of Omicron patients.

Figure 2 and Figure 3 demonstrate the distance measures. Euclidean distance, Hamming distance and Hausdorff distance; all show a decline in value, which indicates the health improvement of patients. These graphs actually display the step-wise treatment of patients. The similar behaviour of all distance measures clearly indicates that the treatment of Omicron patients is going well.

### 5.5. Comparative Study

The proposed model (iv-FHSS) is more flexible and more general as compared to existing structures in following manner:

1. If sub-attributes are replaced with attributes, the model will represent ivfs-set.
2. If intervals are replaced with fuzzy values, the model will represent fhs-set.
3. If the parametrization tool is neglected with attributive sets instead of disjoint attributive valued sets, the model will represent ivf-set.

The proposed model is compared with existing models and illustrated in Table 2:

The following abbreviations are used in Table 2

MF = membership function, SAAF = single argument approximate function, MAAF = multi-argument approximate function, SP = set of parameters, Z= universal set, P(Z)= power set of universe, C(Z)= collection of fuzzy sets, I([0,1])= set of all sub-intervals of [0,1], CP= Cartesian product of disjoint-attributive-valued sets.

## 6. Conclusions

In this study, the concept of iv-FHSS was developed and some new operations such as addition, multiplication, union, intersection, partial membership and partial non-membership for iv-FHSSs were discussed. Euclidean, Hamming and Hausdorff distances for iv-FHSS are discussed. A decision-making algorithm with the support of distance measures based on a real-world application for the treatment of Omicron patients was discussed. Improvement in health of Omicron patients by three different distance measures with pictorial representation was also carried out. Future work for multi-argument approximate function under soft set environments may include hybridized study of structures such as the intuitionistic fuzzy set, neutrosophic set, picture fuzzy set, refined fuzzy set, and pythagorean fuzzy set with interval-valued fuzzy hypersoft set, and their applications in decision making.

## Figures and Tables

**Table 1:** ivfs-set $\left({\tilde{F}}_{S},E\right)$.

Z	$\theta_{1}$	$\theta_{2}$	$\theta_{3}$	$\theta_{4}$	$\theta_{5}$
$z_{1}$	[0.3,0.6]	[0.4,0.5]	[0.7,0.8]	[0.5,0.8]	[0.4,0.6]
$z_{2}$	[0.2,0.4]	[0.3,0.7]	[0.5,0.7]	[0.4,0.5]	[0.5,0.7]
$z_{3}$	[0.3,0.4]	[0.7,0.8]	[0.6,0.8]	[0.5,0.7]	[0.4,0.7]
$z_{4}$	[0.2,0.5]	[0.5,0.7]	[0.6,0.7]	[0.5,0.6]	[0.6,0.8]
$z_{5}$	[0.5,0.6]	[0.4,0.7]	[0.5,0.8]	[0.7,0.8]	[0.3,0.5]
$z_{6}$	[0.4,0.5]	[0.3,0.4]	[0.5,0.6]	[0.4,0.6]	[0.6,0.7]

**Table 2:** Comparative study.

Author	Structure	Function	Domain Set	Range Set	Remarks
Zadeh [3]	*f*-set	MF	Z	[0,1]	Insufficient
Molodtsov [4]	*s*-set	SAAF	SP	P(Z)	Insufficient
Maji [5]	fs-set	SAAF	SP	C(Z)	Insufficient
Yang et al. [6]	ivfs-set	SAAF	SP	P(Z)	Insufficient
Gorzałczany [7]	ivf-set	SAAF	Z	I([0,1])	Insufficient
Smarandache [17]	Hypersoft set	MAAF	CP	P(Z)	Insufficient
Arshad et al.	Proposed structure	MAAF	CP	P(Z)	Sufficient

## Data Availability

Not applicable.
